# Iron competition triggers antibiotic biosynthesis in *Streptomyces coelicolor* during coculture with *Myxococcus xanthus*

**DOI:** 10.1038/s41396-020-0594-6

**Published:** 2020-01-28

**Authors:** Namil Lee, Woori Kim, Jinkyoo Chung, Yongjae Lee, Suhyung Cho, Kyoung-Soon Jang, Sun Chang Kim, Bernhard Palsson, Byung-Kwan Cho

**Affiliations:** 1 Department of Biological Sciences and KI for the BioCentury, Korea Advanced Institute of Science and Technology, Daejeon, 34141 Republic of Korea; 2 Biomedical Omics Group, Korea Basic Science Institute, Cheongju, 28119 Republic of Korea; 3 Division of Bio-Analytical Science, University of Science and Technology, Daejeon, 34113 Republic of Korea; 4 Intelligent Synthetic Biology Center, Daejeon, 34141 Republic of Korea; 5 Department of Bioengineering, University of California San Diego, La Jolla, CA 92093 USA; 6 Department of Pediatrics, University of California San Diego, La Jolla, CA 92093 USA; 7 Novo Nordisk Foundation Center for Biosustainability, Technical University of Denmark, Lyngby, 2800 Denmark

**Keywords:** Next-generation sequencing, Bacteria

## Abstract

Microbial coculture to mimic the ecological habitat has been suggested as an approach to elucidate the effect of microbial interaction on secondary metabolite biosynthesis of *Streptomyces*. However, because of chemical complexity during coculture, underlying mechanisms are largely unknown. Here, we found that iron competition triggered antibiotic biosynthesis in *Streptomyces coelicolor* during coculture with *Myxococcus xanthus*. During coculture, *M. xanthus* enhanced the production of a siderophore, myxochelin, leading *M. xanthus* to dominate iron scavenging and *S. coelicolor* to experience iron-restricted conditions. This chemical competition, but not physical contact, activated the actinorhodin biosynthetic gene cluster and the branched-chain amino acid degradation pathway which imply the potential to produce precursors, along with activation of a novel actinorhodin export system. Furthermore, we found that iron restriction increased the expression of 21 secondary metabolite biosynthetic gene clusters (smBGCs) in other *Streptomyces* species. These findings suggested that the availability for key ions stimulates specific smBGCs, which had the potential to enhance secondary metabolite biosynthesis in *Streptomyces*.

## Introduction

*Streptomyces*, ubiquitous soil gram-positive bacteria, are well-known for their ability to produce diverse secondary metabolites, including many compounds that are currently in clinical use [1]. Functions of secondary metabolites are not directly involved in the natural growth of *Streptomyces* in their native habitats, but they serve important ecological roles in interspecies interaction and communication [2]. Biosynthesis of each secondary metabolite is tightly controlled by the complex regulatory networks in response to various biotic stresses (e.g., nutrient competition, quorum sensing, and physical interaction) and abiotic stresses (e.g., pressure, temperature, and salinity) in natural environments [3]. Under the axenic laboratory culture condition, however, those environmental stimuli are lacking; thus, most of secondary metabolite biosynthetic gene clusters (smBGCs) in *Streptomyces* are not activated [4].

Elucidation of signals that trigger the biosynthesis of the secondary metabolites enables to understand the ecological role of secondary metabolites in the microbial community and increase the industrial production of secondary metabolites. Thus, tremendous efforts have focused on awakening the silent *Streptomyces* smBGCs, including media optimization, epigenetic modifier treatment, mutagenesis, and genetic engineering [5–7]. In addition to these approaches, although accurately mimicking natural habitats is challenging, microbial coculture of two or more different microorganisms in which bacterial populations cohabitate with complex communities, is an effective approach to understand the ecological roles of the chemical diversity of *Streptomyces* and to turn on their cryptic pathways [8, 9]. However, because of the chemical and molecular complexity of microbial communication, success with revealing the interaction mechanisms has been limited. For example, *Myxococcus xanthus* is a mobile predator that is able to lyse a wide variety of bacteria, including *Streptomyces* [10]. Specifically, the interaction between *M. xanthus* and *S. coelicolor* stimulates aerial mycelium formation and actinorhodin overproduction by *S. coelicolor* at the contact area between the two species, but still, the underlying mechanism is ambiguous [11]. In this study, we aimed to elucidate the mechanisms of interaction of these two bacteria during coculture in order to provide insights into actinorhodin biosynthesis and its ecological role. Our findings supported the hypothesis that chemical competition with nearby microbes plays a critical role in stimulating secondary metabolite biosynthesis in *Streptomyces*. An iron-restricted condition triggered the expression of 21 smBGCs in eight *Streptomyces* species, indicating that secondary metabolites involved in different types of microbial communications and interactions have common environmental factors triggering their expression. Precise characterization of the microbial interaction governed by secondary metabolites will uncover the unexplored ecological systems and provide accurate selection pressure for improving the industrial production of secondary metabolites.

## Materials and methods

### Bacterial strains and media

*S. coelicolor* A3(2) M145 (ATCC BAA-471) and seven *Streptomyces* species (*S. subrutilus*, *S. kanamyceticus*, *S. coeruleorubidus*, *S. cinereoruber*, *S. roseosporus*, *S. rimosus*, and *S. venezuelae*) were cultured in 50 mL liquid CTT media (1% Difco casitone, 10 mM Tris-HCl (pH 7.6), 8 mM MgSO_4_, and 1 mM KHPO_4_) with 0.16 g/mL glass beads (3 mm ± 0.3 mm diameter) for 24 h at 30 °C using a 200 rpm orbital shaker. In parallel, *M. xanthus* DK1622 was cultured under the identical conditions used for *Streptomyces* species without the glass beads. Cells were harvested at exponential growth phase (OD_600nm_ between 2 and 3) and resuspended in 1 mL of 20% glycerol for inoculation on solid CTT media. For additional iron treatment, 100 mM FeCl_2_ stock was added to the media at the desired concentration. For iron-restricted conditions, media were supplemented with 10 mM of 2,2′-bipyridyl stock to the desired concentration (350 μM for *S. coelicolor* and 250 μM for seven other *Streptomyces* species to avoid growth retardation under the same conditions as for *S. coelicolor*).

### Solid cocultures

**Fig. 1:**
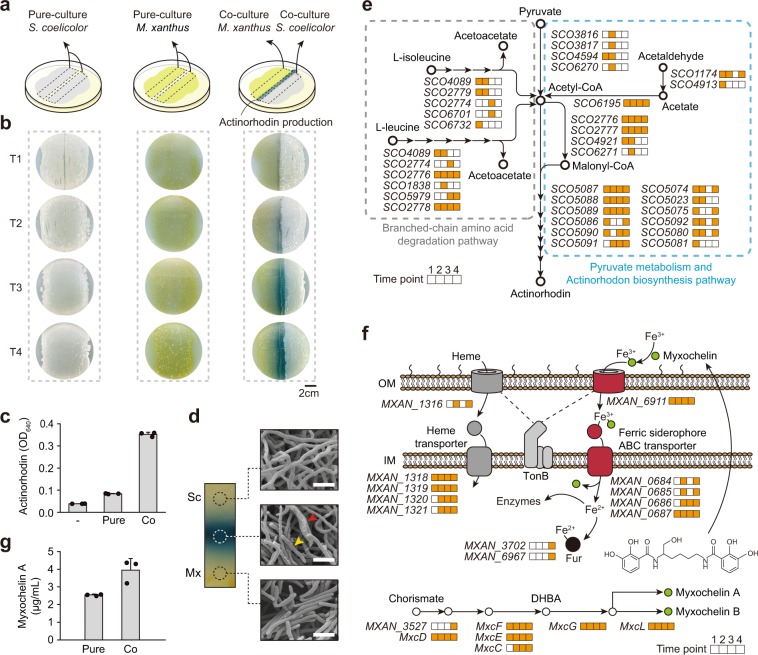
Transcriptome analysis of *S. coelicolor* and *M. xanthus* under pure-culture and coculture conditions. **a** Coculture of *S. coelicolor* and *M. xanthus*. Solid CTT agar plates were inoculated with a lawn of *M. xanthus* and *S. coelicolor*. For pure-culture samples, *S. coelicolor* and *M. xanthus* were spread the same way but with identical species. **b** RNA-Seq sampling was performed after 3 (T1), 5 (T2), 7 (T3), and 9 days (T4) after inoculation. T1 was immediately after *M. xanthus* contacted *S. coelicolor*. **c** Actinorhodin measurement for pure-cultured and cocultured *S. coelicolor*. Error bars indicate standard deviation (s.d.) of three biological replicates. **d** Morphology of *S. coelicolor* and *M. xanthus* during coculture. Cell morphology was determined at the last time point (T4) using scanning electron microscopy (SEM). The yellow arrow indicates *M. xanthus* cells, and the red arrow indicates abnormal hyphae of *S. coelicolor*. Bar: 2 μm. Mx *M. xanthus*, Sc *S. coelicolor*. **e** Upregulated genes involved in the actinorhodin biosynthesis pathway. The four boxes next to the gene name denote T1–4. The orange boxes indicate that genes showing statistically significant upregulation (*p* < 0.05) at the corresponding time point. **f** Upregulated genes involved in the myxochelin biosynthesis pathway and iron acquisition system. Gray and red cylinders represent outer membrane receptors associated with heme and siderophore, respectively. Gray- and red-colored circles indicate periplasmic iron complex binding protein. Gray and red rounded rectangles indicate associations of the iron ferric compound ABC transporter with heme and siderophore, respectively. Yellow–green circles represent myxochelin. DHBA dihydroxybenzoic acid, OM outer membrane, IM inner membrane. **g** Myxochelin A production of pure-cultured and cocultured *M. xanthus*. Error bars indicate standard deviation (s.d.) of three biological replicates.

Cocultures of *S. coelicolor* as a prey with *M. xanthus* as a predator were carried out on solid CTT medium plates. Twenty microliters *S. coelicolor* stock was spread on half (width = 3 cm, length = 6 cm) of the solid CTT medium plate. *M. xanthus* was then spread on the other half, maintaining 3 mm distance from the section of *S. coelicolor*, whereas identical species was spread for pure-culture condition (Fig. 1a). The culture plates were then incubated at 30 °C until the desired morphology appeared. Morphology observation of *S. coelicolor* and *M. xanthus* during coculture using scanning electron microscopy described in Supplementary Methods.

### Genome sequencing for six *Streptomyces* species

Among the eight *Streptomyces* species used in this study, the complete genome sequences of only two species (*S. coelicolor* [NC_003888] and *S. venezuelae* [NC_018750]) were reported. Briefly, genomic DNA (gDNA) from each species was prepared from cultured cells harvested at the mid-exponential phase using a Wizard Genomic DNA Purification Kit (Promega, Madison, WI, USA) following the manufacturer’s instructions. For long-read (PacBio) genome sequencing, a library was prepared using 5 μg gDNA with SMRTbell, according to the manufacturer’s protocol. The prepared SMRTbell library (20 kb size) was sequenced using P6-C4-chemistry (DNA Sequencing Reagent 4.0) on a PacBio RS II sequencing platform (Pacific Biosciences, Menlo Park, CA, USA). For generation of a short-read (Illumina Inc., San Diego, CA, USA) genome sequencing library, gDNA was fragmented to ~350 bp using a Covaris instrument (Covaris Inc., Woburn, MA) with the following conditions: power, 175; duty factor, 20%; C. burst, 200; time, 23 s. The library was then constructed using a TruSeq DNA PCR-Free LT kit (Illumina) according to the manufacturer’s protocol. Finally, prepared short-read genome sequencing libraries were sequenced on a Hiseq 2500 instrument with a 100-bp single-read running scale.

Obtained long-read and short-read genome sequencing read were de novo assembled into contigs and finally linked into chromosome (Supplementary Table 1). Additional details regarding de novo assembly are described in detail in Supplementary Methods. The six completed genomes were annotated using NCBI’s Prokaryotic Genome Annotation Pipeline version 4.5. Genome sequences are available at NCBI under BioProject ID PRJNA412292 (accession numbers: CP023701 [*S. subrutilus*], CP023699 [*S. kanamyceticus*], CP023694 [*S. coeruleorubidus*], CP023693 [*S. cinereoruber*], PDCL00000000 [*S. roseosporus*], and CP023688 [*S. rimosus*]). smBGCs were predicted using each Genbank file by antiSMASH 4.0 (Supplementary Data 3) [12].

### RNA sequencing (RNA-Seq)

For *Streptomyces* species, RNA was extracted from the cells which were harvested from the culture plates and resuspended in 3 mL lysis buffer (20 mM Tris-HCl [pH 7.4], 140 mM NaCl, 5 mM MgCl_2_, and 1% Triton X-100). For *M. xanthus*, RNA was extracted from the cells obtained from the culture plate and resuspended in 3 mL liquid CTT medium. Detailed RNA extraction method is described in Supplementary Methods. RNA-Seq libraries were then constructed using a TruSeq Stranded mRNA LT Sample Prep Kit (Illumina) according to the manufacturer’s instructions. The RNA-Seq library concentration was measured using a Qubit dsDNA HS Assay Kit (Thermo Fisher Scientific Inc., Waltham, MA, USA), and the size of the libraries was determined with an Agilent 2200 TapeStation system (Agilent Technologies, Santa Clara, CA, USA). Quantified libraries were then sequenced on a HiSeq2000 Rapid-Run platform (Illumina) using a 1 × 100 cycle V4 kit.

### Data processing

Sequencing reads were mapped to the corresponding reference genome sequences (Genome accession numbers described above for *Streptomyces* species and NC_008095 for *M. xanthus*) using CLC Genomics Workbench software (CLC Bio, Aarhus, Denmark) (Supplementary Tables 2 and 3). Mapped reads were then counted and normalized using the DESeq2 algorithm in R (Supplementary Data 1–2 and 4–11) [13]. Mapped read information was exported as a BAM file format, which was further converted to a GFF file format containing read counts for each genomic position. The GFF file was visualized on SignalMap (v2.0.0.5; Roche NimbleGen, Basel, Switzerland). The sequencing data were deposited in the European Nucleotide Archive (accession number: PRJEB25075).

### Measurement of iron concentration

Extracellular and intracellular iron level of *S. coelicolor* and *M. xanthus* were measured using an Iron Bio kit for Cedex Bio (Roche Diagnostics, Indianapolis, IN, USA) [14]. Methods for sample preparation is fully descried in Supplementary Methods.

### Measurement of actinorhodin concentrations

Two volumes of methanol were added to the cocultured *S. coelicolor* and solid agar medium (width = 1.5 cm, length = 6 cm) obtained from the culture plates. After overnight incubation at 25 °C, the supernatant was then concentrated in 500 μL methanol by air drying, and 166 μL of 4 N KOH was added to the sample. The concentration of actinorhodin was then measured by UV absorbance at 640 nm with a Tecan Infinite F200 Pro (Tecan Group Lt., Männedorf, Switzerland).

### LC-electrospray ionization (ESI)-MS/MS analysis of myxochelin A

For myxochelin A extraction, two volumes of methanol were added to the cocultured *M. xanthus* and solid agar media obtained from the contact area (width = 1.5 cm, length = 6 cm) in the culture plates. Samples were incubated at 25 °C overnight and supernatant was air-dried and resuspended in 1 mL methanol, which was then analyzed using a Triple Quad 3500 (SCIEX, Framingham, MA, USA) equipped with an ESI source and a Nexera X2 UHPLC system (Shimadzu, Japan). LC-ESI-MS/MS analysis method is described in detail in Supplementary Methods.

### Disruption and overexpression of SCO6666 in *S. coelicolor*

Disruption of SCO6666 gene was performed using CRISPR/Cas9 system based on pCRISPOmyces-2 plasmid following the established protocol [15]. Gene disruption was confirmed by PCR amplification of the genomic region containing *SCO6666* gene. Since the size of *SCO6666* gene is 2.2 kb, the amplified DNA fragment sizes of WT and the deleted strain were 4.8 and 2.6 kb, respectively. Primer sequences used in this validation experiment are listed in Supplementary Table 4. Overexpression of *SCO6666* gene under *ermE* promoter and two endogenous promoters of *S. coelicolor* was conducted by using piBR25 plasmid. Vector construction and transformation method are fully descried in Supplementary Methods.

## Results

### Interspecies interactions between *S. coelicolor* and *M. xanthus*

To compare the effect of interspecies and intraspecies interaction on actinorhodin production of *S. coelicolor*, we formed cocultures by spreading *S. coelicolor* and *M. xanthus* on solid CTT media with a distance of 3 mm between them. For pure-cultures, each species was spread near itself in the same manner as the coculture (Fig. 1a). The growth of *S. coelicolor* was accompanied by the formation of white aerial hyphae. However, in the contact region with *M. xanthus*, *S. coelicolor* showed a glossy morphology. The contact region then widened with growth, and actinorhodin production was observed (Fig. 1b). Only the cocultured *S. coelicolor* produced and exported a large amount of actinorhodin, which was ~6.9 times higher than the pure-cultured *S. coelicolor* (Fig. 1c). Morphologically, *S. coelicolor* interacting with *M. xanthus* appeared to form swollen hyphae, similar to the morphology of lysozyme-treated *S. coelicolor* (Fig. 1d) [11, 16]. This result was related to the observation that *M. xanthus* secretes various enzymes or secondary metabolites with hydrolytic activity when *M. xanthus* preys on vulnerable microbes [17]. *M. xanthus* in the contact region showed the same shape as in the noncontact region and in pure-culture conditions. However, the cell density of *M. xanthus* in the contact region was much lower than in the noncontact region. These results represent that each organism is taking a different strategy during the inter- and intra-species interaction.

### Functional analysis of the differentially expressed genes (DEGs) during coculture

To understand how the cellular processes of the two species were altered during coculture, we performed RNA-Seq using total RNA isolated from cells harvested from the boundary of the contact region at the four time points (Fig. 1a, Supplementary Data 1, and 2, Supplementary Figs. 1, and  2). Among 7,767 protein-coding genes of *S. coelicolor*, ~19% of genes were DEGs between pure-culture and coculture conditions at each of the four time points (*P* < 0.05). In the case of *M. xanthus*, among 7247 genes, ~16% of genes were DEGs during coculture relative to those during pure-culture (Supplementary Fig. 3). The enriched biological function categories of DEGs were screened using ClueGO with Kyoto Encyclopedia of Genes and Genomes pathway terms (*P* < 0.05) [18]. Significantly enriched pathways were detected only among upregulated DEGs in both *S. coelicolor* and *M. xanthus*, but not among downregulated DEGs (Supplementary Fig. 4a–d).

Type II polyketide backbone biosynthesis and branched-chain amino acid degradation pathways in *S. coelicolor* were significantly activated at all coculture time points (Supplementary Fig. 4a). After analyzing the DEG data in detail, we found that the following pathways were activated during coculture which implies the potential to produce key precursors (acetyl-CoA and malonyl-CoA) for actinorhodin biosynthesis: (i) degradation of branched-chain amino acids accumulated acetyl-CoA; (ii) the acetyl-CoA synthetase (SCO6195) converted acetate to acetyl-CoA; and (iii) acetyl-CoA carboxylases (SCO2776, SCO2777, and SCO4921) converted acetyl-CoA to malonyl-CoA (Fig. 1e). Considering that the transcription levels of genes involved in other acetyl-CoA-associated pathways, such as glycolysis and the citrate cycle, showed no differences between pure-culture and coculture conditions (Supplementary Fig. 5), increased acetyl-CoA may be utilized as a precursor for actinorhodin biosynthesis, which is robustly upregulated in coculture condition. Indeed, the branched-chain amino acid catabolism has been reported as the main source for providing the precursor for actinorhodin biosynthesis [19, 20].

Simultaneously, in the case of *M. xanthus*, nine of the 13 upregulated genes at all four time points were related to the iron acquisition system (Supplementary Fig. 4b). Because iron is an essential element for a variety of cellular processes in most living organisms, upregulation of the iron uptake system can be a general cellular response during coculture to occupy a favorable position for iron competition against other bacteria [21]. A representative iron-chelating compound in *M. xanthus* is myxochelin, which is a siderophore released under iron-deficient conditions to form a complex with iron [22]. Then, specific membrane proteins recognize and transport the ferric complex to the cytoplasm, followed by transferring Fe^2+^ from the ferric complex to various enzymes or transcription factors (Fig. 1f) [23]. Under coculture conditions, most of the genes involved in myxochelin biosynthesis and the myxochelin-mediated iron uptake system were upregulated (Fig. 1f, Supplementary Fig. 6a). Indeed, analysis of *M. xanthus* cell extracts using liquid chromatography tandem mass spectrometry (LC-MS/MS) revealed that the level of myxochelin A under coculture conditions was 1.6-fold higher than that under pure-culture conditions (Fig. 1g, Supplementary Fig. 6b).

Taken together, based on the analysis of transcriptome changes in the two species, we found that *S. coelicolor* enhanced the expression of actinorhodin BGCs and actively transformed branched-chain amino acids into acetyl-CoA, which is a key precursor of actinorhodin biosynthesis, during coculture. In contrast, *M. xanthus* activated the production of myxochelin and the myxochelin-mediated iron acquisition system during coculture. Thus, the two organisms exhibited asymmetric responses during coculture.

### Iron competition triggered actinorhodin biosynthesis of *S. coelicolor* during coculture

**Fig. 2:**
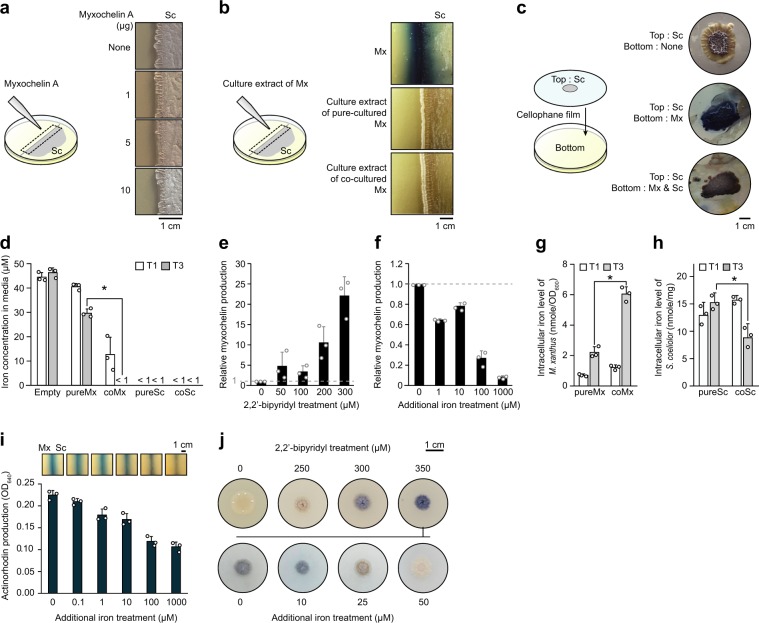
Effects of iron competition between *S. coelicolor* and *M. xanthus* on actinorhodin production. **a** Effects of myxochelin A in pure *S. coelicolor* cultures on actinorhodin production. **b** Actinorhodin production by *S. coelicolor* cultured on CTT agar medium with cell-free extracts of pure-cultured *M. xanthus* and cocultured *M. xanthus*. **c** Effects of physical separation during cocultures using cellophane disks on activation of actinorhodin biosynthesis. **d** Extracellular iron levels remaining in medium after culture. Empty: CTT agar plate without any cell inoculation, cultured under same conditions as other samples; <1 μM: the amount of iron remaining in the medium after culture was lower than the detection limit. Error bars indicate s.d. of three biological replicates. **e** Myxochelin production of pure-cultured *M. xanthus* under iron-restricted conditions. Error bars indicate s.d. of three biological replicates. **f** Changes in myxochelin production during coculture according to iron concentration. Error bars indicate s.d. of three biological replicates. **g** Intracellular iron levels in *M. xanthus*, normalized to the OD_600nm_ of each sample. Error bars indicate s.d. of three biological replicates. **h** Intracellular iron levels in *S. coelicolor*, normalized to the weight of each sample. Error bars indicate s.d. of three biological replicates. **i** Actinorhodin production during coculture according to the iron concentration. Total actinorhodin was extracted from cells and medium and treated with KOH. Absorption at OD_640nm_ indicated actinorhodin production. Error bars indicate s.d. of three biological replicates. **j** Actinorhodin production by pure-cultured *S. coelicolor* under iron-restricted conditions. The upper four colonies were cultured on CTT medium treated with 2,2′-bipyridyl. The lower four colonies were cultured on CTT medium treated with 350 μM 2,2′-bipyridyl and additional iron. Co coculture, Pure pure culture, Sc *S. coelicolor*, Mx *M. xanthus.*

Notably, during coculture, enhanced production of actinorhodin by *S. coelicolor* and myxochelin by *M. xanthus* was observed. However, no enhancement of actinorhodin production by *S. coelicolor* was found following the addition of myxochelin A or cell-free extracts from the pure-cultured and cocultured *M. xanthus* (Fig. 2a, b). In addition, in order to test the effect of biological interaction between two microbes on actinorhodin production, physically separated coculture was performed, resulting in that *S. coelicolor* actively produced actinorhodin without physical contact between the two species (Fig. 2c). These results indicated that cellular chemical compounds, proteins, and physical contact between two microbes did not directly trigger actinorhodin biosynthesis, but the actinorhodin production appears to be stimulated only during the chemical interaction between *M. xanthus* and *S. coelicolor*. Based on these results, we speculated that sequential chemical interaction between the two species, including myxochelin-mediated iron uptake by *M. xanthus*, triggered the actinorhodin biosynthesis of *S. coelicolor* during coculture. Indeed, myxochelin is a catecholate-type siderophore that is a stronger iron-chelating compound than the hydroxamate-type siderophore of *S. coelicolor*, desferrioxamine. Accordingly, *M. xanthus* is capable of fully utilizing iron available in the culture medium, whereas *S. coelicolor* senses reduced iron level followed by the initiation of actinorhodin biosynthesis [24, 25].

To address the iron competition between the two species, we measured extracellular and intracellular iron levels. Even at the T1 time point of *S. coelicolor* under both pure-culture and coculture conditions, the extracellular iron levels, i.e., the amount of iron remaining in the media, decreased lower than the detection limit (<1 μM; Fig. 2d). Owing to the active iron acquisition of *S. coelicolor*, the extracellular iron level of cocultured *M. xanthus* decreased about 6.4-fold more rapidly than that of the pure-cultured *M. xanthus*, indicating the difference in iron requirements of two microbes (Fig. 2d). To respond to the iron-reduced environment caused by *S. coelicolor*, *M. xanthus* overproduced myxochelin. Myxochelin biosynthetic genes (MXAN_3646-3640) possesses a ferric uptake regulator (Fur) binding motif in the upstream region (Supplementary Fig. 6c). Similar to Fur-mediated regulation of the biosynthesis of enterobactin, a siderophore of *Escherichia coli*, myxochelin biosynthesis is activated when Fur is unbound from the promoter region under iron-depletion conditions [26]. Indeed, myxochelin production was increased under iron-restricted pure-culture conditions but decreased under iron-added coculture conditions (Fig. 2e, f).

We then examined changes in intracellular iron levels during the coculture. The intracellular iron concentration of cocultured *M. xanthus* at T3 was about two times higher than that of pure-cultured *M. xanthus*, supporting that the overproduction of myxochelin during coculture increased intracellular iron levels via iron transportation (Fig. 2g). *M. xanthus* apparently sequesters more iron than it needs for the growth during the coculture condition, to prevent *S. coelicolor* from utilizing the iron. Considering the low extracellular iron levels under the coculture conditions, *M. xanthus* seemed to absorb iron at the expense of *S. coelicolor* ability to maintain intracellular iron to high levels. Indeed, in contrast to *M. xanthus*, intracellular iron levels in cocultured *S. coelicolor* were 1.7 times lower than under pure-culture conditions at T3 (Fig. 2h). These results demonstrate that iron competition occurred between the two species during the coculture, when *S. coelicolor* was in a state of the reduced intracellular iron level and stimulated actinorhodin overproduction.

Next, we sought to determine the relationships between iron availability and actinorhodin production. As the amount of iron in the media increased, the actinorhodin production of *S. coelicolor* was decreased during coculture without growth inhibition (Fig. 2i and Supplementary Fig. 7). In addition, we tested whether actinorhodin overproduction was affected by other metal ions, such as divalent cations, including Ca^2+^, Cu^2+^, Zn^2+^, Ni^2+^, Mn^2+^, Mg^2+^, and Co^2+^. In most cases, a high concentration of metal ions inhibited cell growth of *S. coelicolor* or *M. xanthus*. After accounting the cytotoxicity, iron was the only metal ion that decreases actinorhodin production with increased concentrations (Supplementary Figs. 7, 8) [27]. The supplementation of excess iron seems to reduce the interspecies iron competition between *M. xanthus* and *S. coelicolor* and decrease actinorhodin production. Nevertheless, the addition of iron during coculture may have a variety of effects on microbial interaction, so these results are insufficient to explain the direct relationship between actinorhodin production and iron availability. Therefore, we next examined whether actinorhodin production in *S. coelicolor* was triggered in iron-restricted pure-culture conditions by the addition of the iron chelator 2,2′-bipyridyl. *S. coelicolor* produced actinorhodin when the 2,2′-bipyridyl concentration was 300 μM or higher (Fig. 2j). When we added more iron after the 2,2′-bipyridyl treatment, actinorhodin production was no longer induced. These results confirm that the iron-restricted environment induces actinorhodin production in *S. coelicolor*. However, actinorhodin production under the iron-restricted condition was less active than the coculture condition, indicating the existence of another interspecies interaction during coculture, which intensifies the actinorhodin production. Taken together, these results suggest that during coculture, *S. coelicolor* actively took up extracellular iron, resulting in sensing of decreased extracellular iron levels by *M. xanthus* and upregulation of myxochelin biosynthesis. Through the myxochelin-mediated iron acquisition, *M. xanthus* restored intracellular iron levels, which caused *S. coelicolor* to experience iron-restricted conditions and induce actinorhodin production. Also, actinorhodin production seems to be further activated by another interspecies interaction during coculture.

### Effects of iron depletion on the expression of smBGCs in *Streptomycetes*

We then examined whether iron-restricted conditions altered the expression of smBGCs in other *Streptomycetes*. Seven species (i.e., *S. subrutilus*, *S. kanamyceticus*, *S. coeruleorubidus*, *S. cinereoruber*, *S. roseosporus*, *S. rimosus*, and *S. venezuelae*) were selected based on morphological changes, such as colony shape and color, under iron-restricted conditions (Supplementary Fig. 9). We completed the genome sequences of six species in a single scaffold (7.6–10.1 Mbp in length) for five species and two scaffolds (5.7 and 2.1 Mbp in length) for *S. roseosporus* using both long-read (PacBio) and short-read (Illumina) genome sequencing methods (Supplementary Fig. 10 and Supplementary Table 1). The completed genomes were then annotated by the NCBI Prokaryotic Genome Annotation Pipeline and smBGCs were predicted using antiSMASH [12]. Finally, 260 smBGCs were predicted from the eight *Streptomycetes* genomes (including *S. coelicolor*; ~30 smBGCs/genome; Supplementary Data 3).

**Fig. 3:**
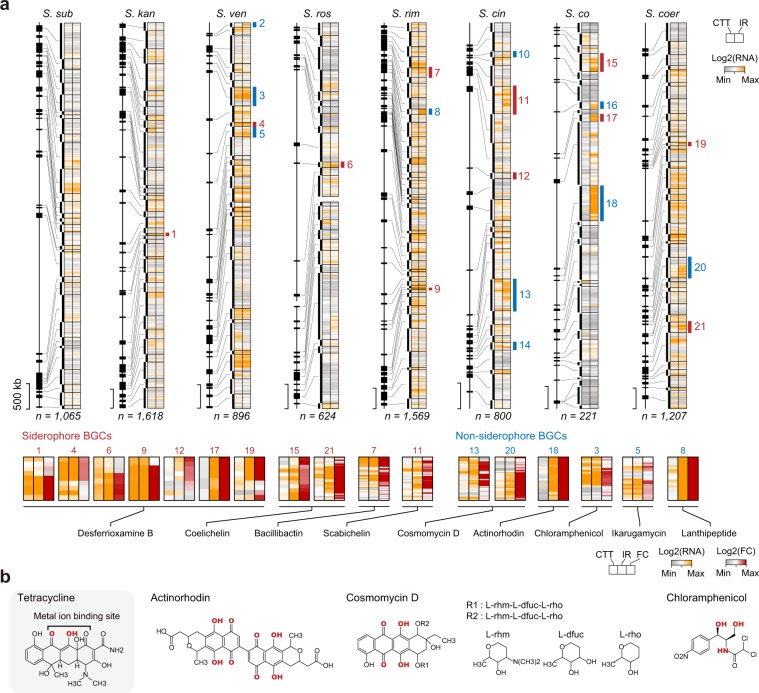
Activated smBGCs from eight *Streptomyces* species under iron-restricted culture conditions. **a** Transcriptional landscape of 260 smBGCs in eight *Streptomyces* species under iron-restricted conditions. The heat maps show log_2_ expression values of genes in smBGCs. The left side of each heat map represents the position of smBGCs in the sequenced genome. If the median of the normalized expression value of genes in smBGC under iron-restricted conditions was higher than that of actinorhodin BGCs under coculture conditions (341.46), the smBGC was assigned as an expressed smBGC. If the median of log_2_ fold change value of smBGC-encoded genes was higher than that of actinorhodin BGC under coculture conditions (0.71), the smBGC was assigned as an upregulated smBGC under iron-restricted conditions. Eleven upregulated siderophore BGCs are shown in red, whereas ten upregulated nonsiderophore BGCs are shown in blue. The enlarged heat map shows the log_2_ expression and log_2_ fold change values of the 11 upregulated siderophore BGCs and six nonsiderophore BGC-encoded genes, excluding ectoine and melanin BGCs. *S. sub* *S. subrutilus*, *S. kan* *S. kanamyceticus*, *S. ven* *S. venezuelae*, *S. ros* *S. roseosporus*, *S. rim* *S. rimosus*, *S. cin* *S. cinereoruber*, *S. co* *S. coelicolor*; *S. coer* *S. coeruleorubidus*, CTT pure-cultured in CTT solid medium, IR pure-cultured in iron-restricted CTT solid medium, FC fold change of RNA expression value. **b** Chemical structures of secondary metabolites produced from the six nonsiderophore BGCs upregulated under iron-restricted conditions. Red-colored chemical residues represent putative interacting sites with metal ions.

To determine which smBGCs were activated under iron-restricted conditions, we then performed RNA-Seq under iron-restricted (2,2′-bipyridyl-treated CTT media) and normal (CTT media) conditions (Supplementary Table 3). RNA-Seq reads were mapped to the corresponding completed genomes, and the expression value of each gene was normalized using the DESeq2 package in R (Supplementary Data 4–11) [13]. Calculation of Euclidean distances between samples demonstrated high reproducibility between biological replicates (Supplementary Fig. 11). Among 260 smBGCs predicted in the genomes of the eight species, 76 smBGCs were expressed in normal or iron-restricted conditions, and the expression of 21 smBGCs was specifically upregulated in response to iron-restricted conditions (Fig. 3a). Among them, 11 smBGCs were siderophore BGCs, including desferrioxamine, coelichelin, bacillibactin, and scabichelin BGCs (Fig. 3a). Biosynthesis of desferrioxamine and coelichelin in *S. coelicolor* is regulated by the iron-dependent transcriptional regulator DmdR1 in the same way that Fur regulates myxochelin biosynthesis in *M. xanthus* [28, 29]. Consistent with this, the remaining nine siderophore BGCs also contained DmdR1-binding sites, indicating that expression of these siderophore BGCs was regulated by DmdR1 (Table 1).

**Table 1:** DmdR1-binding sites in 11 siderophore BGCs upregulated under iron-restricted conditions.

BGC	BGC number	Species	Gene	DmdR1-binding sites (5′-3′ sequence)
Desferrioxamine B	1	*S. kanamyceticus*	*CP970_27940*	TTAGGTTAGGCTCACCTAA (63 nt) **ATG**
	4	*S. venezuelae*	*SVEN2570*	TTAGGTTAGGCTaACCTAA (50 nt) **ATG**
	6	*S. roseosporus*	*CP979_13235*	TTAGGTTAGGCTaACCTtA (54 nt) **ATG**
	9	*S. rimosus*	*CP984_32060*	TTAGGTTAGGCTtACCTcA (115 nt) **ATG**
	12	*S. cinereoruber*	*CP977_12440*	TTAGGTTAGGCTCACCTAc (68 nt) **ATG**
	17	*S. coelicolor*	*SCO2782*	TTAGGTTAGGCTCACCTAA (63 nt) **ATG**
	19	*S. coeruleorubidus*	*CP976_16210*	TTAGGTTAGGCTtACCTAA (62 nt) **ATG**
Coelichelin	15	*S. coelicolor*	*SCO0498*	TTAGcTTAGGCTCACCTAA (25 nt) **ATG**
			*SCO0490*	TTAGGTTAGGCTCgCCTAA (49 nt) **ATG**
	21	*S. coeruleorubidus*	*CP976_37645*	TgAGcTTAGcCTtACCTAA (104 nt) **ATG**
			*CP976_37695*	TTAGGTTAGGCTCgCCTAA (50 nt) **ATG**
Bacillibactin	7	*S. rimosus*	*CP984_01935*	TaAGGTaAGGCTCgCCTTA (49 nt) **ATG**
			*CP984_01965*	TaAGGTTAGcCTaACCTAA (124 nt) **ATG**
Scabichelin	11	*S. cinereoruber*	*CP977_03980*	TTAGGTTAGGCTaAgCTAA (83 nt) **ATG**
			*CP977_04015*	TTAGGTaAGcCTtACCTAA (101 nt) **ATG**
			*CP977_04025*	TTAGGTaAGcCTtACCTAA (40 nt) **ATG**

Sequences in lower case indicate nonconserved nucleotides compared with the known DmdR1-binding site in the desferrioxamine BGC of *S. coelicolor*.

In contrast, the remaining ten smBGCs, activated by iron restriction, corresponded to the synthesis of various secondary metabolites, including ectoine, melanin, actinorhodin, cosmomycin D, ikarugamycin, chloramphenicol, and lanthipeptide. Among these, ectoine and melanin BGCs were conserved in all eight species, but only upregulated in three and one species, respectively, indicating that these BGCs were differently regulated by iron restriction in each species. The remaining six BGCs were species specific, except cosmomycin D BGC, which was conserved in *S. coeruleorubidus* and *S. cinereoruber* (Fig. 3a). Unlike siderophore BGCs, putative binding sites of Fur or DmdR1 were not detected in these six BGCs. However, a repeat of the TCGAG sequence at constant intervals (6 nt) was found in actinorhodin and cosmomycin D [30]. The motif was likely to be the binding site for the cluster-situated SARP family regulator, which was located in both BGCs (Supplementary Fig. 12). Overall, through the genome sequencing and transcriptome analyses, our findings suggested that siderophore BGCs of *Streptomyces* were activated via DmdR1-associated regulation under iron-restricted conditions. In contrast, nonsiderophore BGCs, including actinorhodin BGC, appeared to be upregulated by other mechanisms that may be associated with iron limiting culture conditions.

Among the secondary metabolites expected to be produced from the six nonsiderophore BGCs, actinorhodin and cosmomycin D were aromatic polyketide-type secondary metabolites with a similar backbone structure (Fig. 3b). Interestingly, this backbone structure (close ketone and hydroxyl groups) is highly similar to the structure of another polyketide, tetracycline which is well-known for chelating Fe^2+^/Fe^3+^ ions [31]. In addition, chloramphenicol, a nonribosomal peptide-type secondary metabolite, has three potential sites for interactions with metal ions, and these sites have been reported to form a complex with Fe ion (Fig. 3b) [32]. Taken together, nonsiderophore secondary metabolites, of which BGCs were upregulated under iron-restricted conditions, have the potential to interact with iron ion. Although further analyses are required, if these secondary metabolites have both antibiotic and iron-chelating functions, production of these secondary metabolites during the iron competition with other microbes would be highly advantageous to *Streptomyces* by blocking the accessibility of other microbes and increasing the iron-chelating ability.

### Identification of novel genes affecting actinorhodin production in *S. coelicolor*

Although iron-restricted culture conditions triggered actinorhodin production, the amount synthesized was markedly lower, and the produced actinorhodin was rarely excreted compared with that under coculture conditions (Fig. 2j). Because changes in the transcriptome of *S. coelicolor* during coculture were related to the sophisticated interaction with *M. xanthus*, iron-restricted conditions were insufficient to mimic the phenotype of coculture. Thus, in order to elucidate the cellular mechanisms that supported actinorhodin production or transport during coculture, we compared the DEGs between coculture and pure-culture conditions with the DEGs between iron-restricted conditions and normal (CTT media) culture condition. Among the 30 upregulated genes during coculture conditions, seven genes were commonly upregulated genes during the iron-restricted condition, simultaneously, four out of eight downregulated genes during coculture conditions were commonly downregulated during the iron-restricted condition (Supplementary Fig. 13). Three of the seven commonly upregulated genes were minimal polyketide synthase-coding genes (SCO5087–5089), which are essential for actinorhodin biosynthesis [19]. However, the branched-chain amino acid degradation pathway, which was upregulated during coculture and potentially supplies precursors for actinorhodin biosynthesis, was not upregulated under iron-restricted conditions. This was thought to be one of the reasons why less actinorhodin was produced under iron-restricted conditions compared with coculture conditions.

**Fig. 4:**
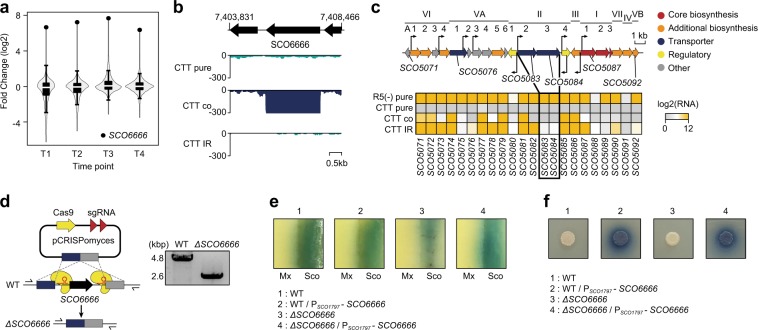
Iron-independent transcriptional activation of novel genes involved in actinorhodin production during coculture. **a** A violin and box plot shows the distribution of mRNA expression fold change between coculture and pure-culture conditions according to growth. Box limits, whiskers, center lines indicate 1st and 3rd quartiles, 10 and 90 percentiles, and median of the distribution, respectively. Black closed circled indicates the expression level of SCO6666 gene. **b** RNA expression pattern of the SCO6666 gene under various culture conditions. **c** Differential expression pattern of actinorhodin BGC under various culture conditions. **d** Schematic of the CRISPR/Cas9-based knockout and PCR evaluation of the SCO6666 gene knockout. **e** Phenotype of SCO6666 deletion strains under coculture conditions. **f** Phenotype of the SCO6666-overexpressing strain under pure-culture conditions. WT wild-type, Sco *S. coelicolor*, Mx *M. xanthus*, *ΔSCO6666* SCO6666-deleted *S. coelicolor*, P_*SCO1797*_ – *SCO6666* SCO6666-overexpressing strain with the SCO1797 promoter, CTT pure pure-cultured in CTT solid medium, CTT co cocultured in CTT solid medium, CTT IR pure-cultured in iron-restricted CTT solid medium, R5(−) pure pure-cultured in R5(−) solid medium.

Among genes specifically upregulated during coculture, SCO6666, which had the highest fold change in expression between pure-culture and coculture conditions at all four time points, rarely expressed under iron-restricted conditions (Fig. 4a, b). SCO6666 encodes a hypothetical protein but has a protein sequence similar to that of the actinorhodin BGC-encoded gene SCO5084 (e-value < 3 × 10^−^^5^ and identity = 34%), which has been predicted as a transporter or dehydrogenase to convert actinorhodin to γ-actinorhodin [33]. SCO6666 possesses two mycobacterial membrane proteins large domains and a putative NAD-binding domain. Therefore, we speculated that SCO6666 may play critical roles in actinorhodin production during coculture. Interestingly, SCO5083 and SCO5084 in the actinorhodin BGC were rarely expressed under CTT pure-culture, coculture, and iron-restricted conditions (Fig. 4c). Normally, the two genes are transcriptionally repressed by a repressor encoded by SCO5082 (*actII-ORF1*) and activated when actinorhodin or intermediates bind to the repressor and release it from the promoter region [34]. Because actinorhodin was not produced under the pure-culture conditions, the expression of the two genes may be repressed; in contrast, under actinorhodin-producing conditions, such as coculture and iron-restricted conditions, these two genes were expected to be strongly activated. Therefore, we suspected that there was an additional regulatory mechanism acting on the SCO5083 and SCO5084 genes under CTT media culture conditions. Accordingly, we performed additional RNA-Seq on *S. coelicolor* cultured in R5(−) solid media, the most common culture conditions for *S. coelicolor*. The results revealed that actinorhodin was actively produced and that SCO5083–5084 were highly expressed, as expected (Supplementary Data 4 and Fig. 4c) [35]. The low expression of SCO5083–5084 may be why low actinorhodin production was observed under iron-restricted CTT culture conditions. In contrast, under coculture condition, actinorhodin was still actively produced and exported to the extracellular region, even in the absence of SCO5083–5084 expression. These results suggested that genes, including SCO6666, activated only during coculture had the potential to act as alternative genes to replace SCO5083–SCO5084.

To verify the functional roles of SCO6666 in actinorhodin production or export, a deletion strain was constructed using a CRISPR/Cas9-mediated knockout system (Fig. 4d). The SCO6666 deletion strain did not produce actinorhodin under pure-culture conditions, similar to the wild-type strain. Moreover, the deletion strain produced approximately three times less actinorhodin during coculture compared with the wild-type strain (Fig. 4e). To further elucidate the functional role of this gene, we examined the correlations between SCO6666 expression and actinorhodin production under pure-culture conditions. SCO6666 is a membrane protein that can be toxic to the host if overexpressed [36]. Accordingly, based on the RNA-Seq results, we selected two weak promoters from SCO1797 and SCO1855, which showed constant expression during cell growth (Supplementary Fig. 14a). The constructed SCO6666 overexpression strain with the SCO1797 promoter actively produced actinorhodin under pure-culture conditions and iron-restricted conditions (Fig. 4f). In contrast, actinorhodin production in the SCO6666-overexpressing strain with the SCO1855 promoter, showing tenfold less RNA expression than that with the SCO1797 promoter, was similar to that of the wild-type strain, indicating that SCO6666 expression was insufficient to activate actinorhodin production. Consistent with this, SCO6666 expression levels of the strain with the SCO1797 promoter were 5.73- and 2.66-fold higher than that in the wild-type and the strain with the SCO1855 promoter, respectively (Supplementary Fig. 14b). In addition, the complemented strain constructed by introducing SCO6666 overexpression vector into SCO6666 deletion strain showed the same phenotype as the SCO6666 overexpression strain in coculture and pure-culture conditions (Fig. 4e, f). The fact that actinorhodin export by the SCO6666 deletion strain did not completely disappear under coculture conditions suggested that actinorhodin export during coculture was not entirely mediated by SCO6666. Taken together, we concluded that SCO6666 was activated in an iron-independent manner during coculture conditions and played an important role in actinorhodin production by acting with actinorhodin cluster genes.

## Discussion

Numerous studies aimed to identify the effect of microbial interaction during coculture on secondary metabolite production [37, 38]. Most of these studies have focused on understanding differences in metabolic profiles between coculture and pure-culture conditions using mass spectrometry [39]. However, because of complex interspecies interaction, the underlying mechanisms are still elusive [40–42]. Accordingly, in this study, we used transcriptome analysis, physiological measurements, and functional analysis of genes to show that chemical competition for iron, not physical contact, between the two microbes triggered actinorhodin production of *S. coelicolor* during coculture with *M. xanthus*.

**Fig. 5:**
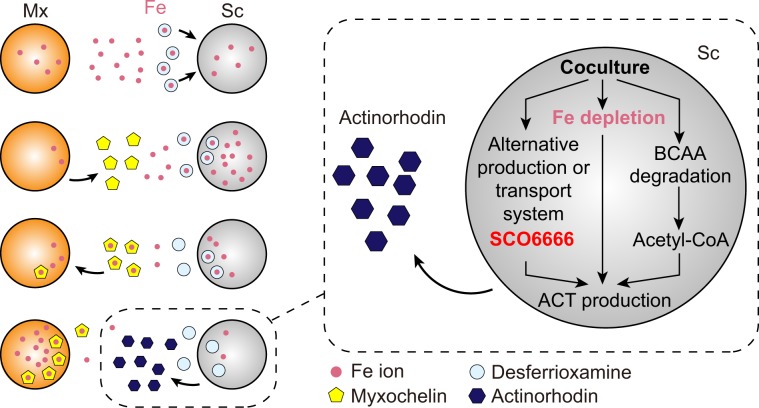
Proposed microbial interaction between *S. coelicolor* and *M. xanthus* during coculture. Sc *S. coelicolor*, Mx *M. xanthus.*

Iron is an essential ingredient for fundamental biological processes, including respiration, DNA synthesis, and translation. Although iron is one of the most abundant elements on Earth, in the environment, iron concentrations in water are extremely low, and iron in the soil is present in an insoluble form, resulting microbes to be prone to experience iron limitation [43]. In order to fulfill iron requirements during competition with neighbors, bacteria increase the expression of genes involved in the iron acquisition system, including the biosynthesis of siderophore [43, 44]. Thus, we speculated that the different iron affinity levels of the two siderophores, myxochelin, and desferrioxamine [24, 25], enable *M. xanthus* to obtain higher levels of iron, which causes *S. coelicolor* to produce actinorhodin in response to iron-reduced conditions (Fig. 5) [45]. Because actinorhodin acts as a repellent to *M. xanthus* and has the structural potential to chelate iron, actinorhodin production appears to support *S. coelicolor* to be in a favorable position in iron competition during coculture [45]. Considering that actinorhodin production is only triggered by the interspecies iron competition, not intraspecies iron competition, it seems to have a selective advantage to tolerate conspecifics but be more aggressive against xeno-species. In the same context, actinorhodin production during the iron-restricted condition is less intense than the coculture condition, indicating that another interspecies interaction between *S. coelicolor* and *M. xanthus* exists which intensifies the actinorhodin production.

Indeed, by comparing the transcriptome data for *S. coelicolor* under coculture and iron-restricted conditions, we found that actinorhodin cluster-related genes as well as expression of other genes affected actinorhodin biosynthesis. Actinorhodin and its intermediates induce the expression of SCO5083–5084 to transport actinorhodin and increase cell resistance to actinorhodin [34]. Surprisingly, our results implied the existence of additional regulatory mechanisms repressing SCO5083–5084 expression during the actinorhodin production. In addition, a knockout and overexpression study of SCO6666 suggested its functional role to replace SCO5083–5084 for actinorhodin biosynthesis. Although further studies are required, assembly of a membrane-associated multiprotein complex, such as SCO5083–5084 and SCO6666, is required for high-level secondary metabolite production [46]. Taken together, actinorhodin production seems to be not only determined by genes present in the actinorhodin cluster but also determined by a more complex network involving other unknown genes (Fig. 5).

In addition, in order to identify the effect of iron depletion on other secondary metabolites production, we performed genome sequencing and transcriptome analyses on seven other *Streptomyces* species, determining that iron-restricted conditions activated 11 siderophore BGCs and 10 nonsiderophore BGCs from eight *Streptomyces* species, including *S. coelicolor*. The 11 siderophore BGCs contained regulatory sites controlled by DmdR1, which was well-conserved in all eight *Streptomyces* species, suggesting that siderophore BGC expression may be regulated by DmdR1 in response to iron levels. In contrast, nonsiderophore BGCs seem to be activated by mechanisms other than Fur- or DmdR1-mediated regulation under iron-restricted conditions. In the environment, siderophores produced by *Streptomyces* are sometimes stolen and utilized by other nearby microbes, such as fungi and other *Streptomyces* species [47]. Therefore, *Streptomyces* may activate defense mechanisms against neighboring species to survive under conditions of iron competition, as in the example of *S. coelicolor* and *M. xanthus*. In the same context, pathogenic bacteria use iron-depletion conditions as a marker for host recognition and virulence expression to take nutrients, including iron, from the host [21]. Accordingly, when *Streptomyces* species reside with various microbes and undergo iron competition, they increase the production of nonsiderophore secondary metabolites, which have antimicrobial activity and potentially iron-chelating function to obtain advantages during competition.

In order to increase the chemical diversity of known secondary metabolites and to search new drug candidates from *Streptomyces*, activating the silent biosynthetic pathways of *Streptomyces* is important, as is improving our understanding the awakening mechanism. Overall, our findings provided insights into the mechanisms through which chemical competition in the microbial community activates the silent secondary biosynthetic pathways in unexpected ways and thus will elevate the potential of *Streptomyces* as a production host for a diverse set of secondary metabolites.

## Supplementary information

Supplementary information

Dataset 1

Dataset 2

Dataset 3

Dataset 4

Dataset 5

Dataset 6

Dataset 7

Dataset 8

Dataset 9

Dataset 10

Dataset 11

## References

[1] Nett M, Ikeda H, Moore BS (2009). Genomic basis for natural product biosynthetic diversity in the actinomycetes. Nat Prod Rep..

[2] O’Brien J, Wright GD (2011). An ecological perspective of microbial secondary metabolism. Curr Opin Biotechnol..

[3] Cihak M, Kamenik Z, Smidova K, Bergman N, Benada O, Kofronova O (2017). Secondary metabolites produced during the germination of *Streptomyces coelicolor*. Front Microbiol..

[4] Antoraz S, Santamaria RI, Diaz M, Sanz D, Rodriguez H (2015). Toward a new focus in antibiotic and drug discovery from the *Streptomyces arsenal*. Front Microbiol.

[5] Scherlach K, Hertweck C (2009). Triggering cryptic natural product biosynthesis in microorganisms. Org Biomol Chem.

[6] Chiang YM, Lee KH, Sanchez JF, Keller NP, Wang CC (2009). Unlocking fungal cryptic natural products. Nat Prod Commun.

[7] Hertweck C (2009). Hidden biosynthetic treasures brought to light. Nat Chem Biol.

[8] Onaka H (2017). Novel antibiotic screening methods to awaken silent or cryptic secondary metabolic pathways in actinomycetes. J Antibiotics.

[9] Marmann A, Aly AH, Lin WH, Wang BG, Proksch P (2014). Co-cultivation-A powerful emerging tool for enhancing the chemical diversity of microorganisms. Mar Drugs..

[10] Berleman JE, Kirby JR (2009). Deciphering the hunting strategy of a bacterial wolfpack. FEMS Microbiol Rev..

[11] Perez J, Munoz-Dorado J, Brana AF, Shimkets LJ, Sevillano L, Santamaria RI (2011). *Myxococcus xanthus* induces actinorhodin overproduction and aerial mycelium formation by *Streptomyces coelicolor*. Micro Biotechnol..

[12] Blin K, Wolf T, Chevrette MG, Lu X, Schwalen CJ, Kautsar SA, et al. AntiSMASH 4.0-improvements in chemistry prediction and gene cluster boundary identification. Nucleic Acids Res. 2017;45:W36–41. 10.1093/nar/gkx319 PMC5570095 28460038

[13] Anders S, Huber W. Differential expression analysis for sequence count data. Genome Biol. 2010;11:R106. 10.1186/gb-2010-11-10-r106 PMC3218662 20979621

[14] Riemer J, Hoepken HH, Czerwinska H, Robinson SR, Dringen R (2004). Colorimetric ferrozine-based assay for the quantitation of iron in cultured cells. Anal Biochem..

[15] Cobb RE, Wang Y, Zhao H (2015). High-efficiency multiplex genome editing of *Streptomyces* species using an engineered CRISPR/Cas system. ACS Synth Biol.

[16] Gray DI, Gooday GW, Prosser JI (1990). Apical hyphal extension in *Streptomyces coelicolor* A3(2). J Gen Microbiol.

[17] Munoz-Dorado J, Marcos-Torres FJ, Garcia-Bravo E, Moraleda-Munoz A, Perez J (2016). Myxobacteria: Moving, Killing, Feeding, and Surviving Together. Front Microbiol.

[18] Bindea G, Mlecnik B, Hackl H, Charoentong P, Tosolini M, Kirilovsky A (2009). ClueGO: a cytoscape plug-in to decipher functionally grouped gene ontology and pathway annotation networks. Bioinformatics..

[19] Ryu YG, Butler MJ, Chater KF, Lee KJ (2006). Engineering of primary carbohydrate metabolism for increased production of actinorhodin in *Streptomyces coelicolor*. Appl Environ Microbiol..

[20] Stirrett K, Denoya C, Westpheling J (2009). Branched-chain amino acid catabolism provides precursors for the Type II polyketide antibiotic, actinorhodin, via pathways that are nutrient dependent. J Ind Microbiol Biotechnol.

[21] Skaar EP (2010). The battle for iron between bacterial pathogens and their vertebrate hosts. PLoS Pathog..

[22] Li Y, Weissman KJ, Muller R (2008). Myxochelin biosynthesis: direct evidence for two- and four-electron reduction of a carrier protein-bound thioester. J Am Chem Soc..

[23] Cornelis P (2010). Iron uptake and metabolism in pseudomonads. Appl Microbiol Biotechnol.

[24] Miethke M, Marahiel MA (2007). Siderophore-based iron acquisition and pathogen control. Microbiol Mol Biol Rev.

[25] Winkelmann Gn. CRC handbook of microbial iron chelates. Boca Raton, USA: CRC Press; 1991. p. 366.

[26] Seo SW, Kim D, Latif H, O’Brien EJ, Szubin R, Palsson BO (2014). Deciphering Fur transcriptional regulatory network highlights its complex role beyond iron metabolism in *Escherichia coli*. Nat Commun..

[27] Ahmed E, Holmstrom SJ (2014). Siderophores in environmental research: roles and applications. Micro Biotechnol..

[28] Cornelis P, Wei Q, Andrews SC, Vinckx T (2011). Iron homeostasis and management of oxidative stress response in bacteria. Metallomics..

[29] Craig M, Lambert S, Jourdan S, Tenconi E, Colson S, Maciejewska M (2012). Unsuspected control of siderophore production by N-acetylglucosamine in streptomycetes. Environ Microbiol Rep.

[30] Arias P, Fernandez-Moreno MA, Malpartida F (1999). Characterization of the pathway-specific positive transcriptional regulator for actinorhodin biosynthesis in *Streptomyces coelicolor* A3(2) as a DNA-binding protein. J Bacteriol..

[31] Bechet M, Blondeau R (1998). Iron deficiency-induced tetracycline production in submerged cultures by *Streptomyces aureofaciens*. J Appl Microbiol..

[32] Refat FAIA-KaMS (2016). Synthesis, spectroscopic, thermal and anticancer studies of metal-antibiotic chelations: Ca(II), Fe(III), Pd(II) and Au(III) chloramphenicolcomplexes. J Mol Struct.

[33] Bystrykh LV, Fernandez-Moreno MA, Herrema JK, Malpartida F, Hopwood DA, Dijkhuizen L (1996). Production of actinorhodin-related “blue pigments” by *Streptomyces coelicolor* A3(2). J Bacteriol.

[34] Xu Y, Willems A, Au-Yeung C, Tahlan K, Nodwell JR (2012). A two-step mechanism for the activation of actinorhodin export and resistance in *Streptomyces coelicolor*. MBio..

[35] Jeong Y, Kim JN, Kim MW, Bucca G, Cho S, Yoon YJ (2016). The dynamic transcriptional and translational landscape of the model antibiotic producer *Streptomyces coelicolor* A3(2). Nat Commun.

[36] Wagner S, Baars L, Ytterberg AJ, Klussmeier A, Wagner CS, Nord O (2007). Consequences of membrane protein overexpression in *Escherichia coli*. Mol Cell Proteom.

[37] Traxler MF, Watrous JD, Alexandrov T, Dorrestein PC, Kolter R (2013). Interspecies interactions stimulate diversification of the *Streptomyces coelicolor* secreted metabolome. MBio..

[38] Ueda K, Kawai S, Ogawa H, Kiyama A, Kubota T, Kawanobe H (2000). Wide distribution of interspecific stimulatory events on antibiotic production and sporulation among *Streptomyces* species. J Antibiot.

[39] Bertrand S, Bohni N, Schnee S, Schumpp O, Gindro K, Wolfender JL (2014). Metabolite induction via microorganism co-culture: A potential way to enhance chemical diversity for drug discovery. Biotechnol Adv.

[40] Luti KJ, Mavituna F (2011). *Streptomyces coelicolor* increases the production of undecylprodigiosin when interacted with *Bacillus subtilis*. Biotechnol Lett..

[41] Luti KJ, Mavituna F (2011). Elicitation of *Streptomyces coelicolor* with dead cells of *Bacillus subtilis* and *Staphylococcus aureus* in a bioreactor increases production of undecylprodigiosin. Appl Microbiol Biotechnol..

[42] Schaberle TF, Orland A, Konig GM (2014). Enhanced production of undecylprodigiosin in *Streptomyces coelicolor* by co-cultivation with the corallopyronin A-producing myxobacterium, *Corallococcus coralloides*. Biotechnol Lett..

[43] Chu BC, Garcia-Herrero A, Johanson TH, Krewulak KD, Lau CK, Peacock RS (2010). Siderophore uptake in bacteria and the battle for iron with the host; a bird’s eye view. Biometals..

[44] Hjerde E, Karlsen C, Sorum H, Parkhill J, Willassen NP, Thomson NR (2015). Co-cultivation and transcriptome sequencing of two co-existing fish pathogens *Moritella viscosa* and *Aliivibrio wodanis*. BMC Genomics.

[45] Coisne S, Bechet M, Blondeau R (1999). Actinorhodin production by *Streptomyces coelicolor* A3(2) in iron-restricted media. Lett Appl Microbiol..

[46] Straight PD, Fischbach MA, Walsh CT, Rudner DZ, Kolter R (2007). A singular enzymatic megacomplex from *Bacillus subtilis*. Proc Natl Acad Sci USA.

[47] Traxler MF, Seyedsayamdost MR, Clardy J, Kolter R (2012). Interspecies modulation of bacterial development through iron competition and siderophore piracy. Mol Microbiol..

