# Supplementary material for: Iron competition triggers antibiotic biosynthesis in Streptomyces coelicolor during coculture with Myxococcus xanthus
Source: ISME J. 2020 Jan 28;14(5):1111–24. doi: 10.1038/s41396-020-0594-6 (PMC7174319; doi:10.1038/s41396-020-0594-6)
Supplement: Supplementary file 1 — Supplementary information [file 41396_2020_594_MOESM1_ESM.docx]

Supplementary Information on

**Iron competition triggers antibiotic biosynthesis in *Streptomyces coelicolor* during co-culture with *Myxococcus xanthus***

Namil Lee^1^, Woori Kim^1^, Jinkyoo Chung^1^, Yongjae Lee^1^, Suhyung Cho^1^, Kyoung-Soon Jang^2,3^, Sun Chang Kim^1,2^, Bernhard Palsson^4,5,6^, and Byung-Kwan Cho^1,7,*^

^1^Department of Biological Sciences and KI for the BioCentury, Korea Advanced Institute of Science and Technology, Daejeon, 34141, Republic of Korea

^2^Biomedical Omics Group, Korea Basic Science Institute, Cheongju, 28119, Republic of Korea

^3^Division of Bio-Analytical Science, University of Science and Technology, Daejeon 34113, Republic of Korea

^4^Department of Bioengineering, University of California San Diego, La Jolla, CA, 92093, USA

^5^Department of Pediatrics, University of California San Diego, La Jolla, CA, 92093, USA

^6^Novo Nordisk Foundation Center for Biosustainability, Technical University of Denmark, Lyngby, 2800, Denmark.

^7^Intelligent Synthetic Biology Center, Daejeon, 34141, Republic of Korea

*Correspondence and request for materials should be addressed to B.K.C. (email: bcho@kaist.ac.kr)

**Supplementary Methods**

***Scanning electron microscopy***

Samples were fixed in 2.5% paraformaldehyde-glutaraldehyde mixture buffered with 0.1 M phosphate (pH 7.2) for 2 h, postfixed in 1% osmium tetroxide in the same buffer for 1 h, dehydrated in graded ethanol, and substituted with isoamyl acetate. Samples were then dried at the critical point in CO_2_, sputtered with gold in a sputter coater (SC502; Polaron) and observed using scanning electron microscopy (FEI Quanta 250 FEG installed in KRIBB).

***De novo genome assembly for six Streptomyces species***

*De novo* assembly of long-read genome sequencing reads was conducted using the HGAP assembly process workflow (Version 2.3), including consensus polishing with Quiver [1]. For short-read genome sequencing reads, initial trimming was performed based on sequence quality using a CLC Genomics Workbench software (CLC Bio, Aarhus, Denmark; parameters: ambiguous trim limit, 2; quality trim limit, 0.05), and reads were then assembled using the *de novo* assembly function of CLC genomic workbench with default settings. Assembled contigs from short-read genome sequencing reads were aligned with one to two assembled contigs from long-read genome sequencing reads using the MAUVE and CLC genomic workbench. We linked contigs and/or extended 5′ and/or 3′ ends using the GAP5 program (Staden package) [2]. After extension of the chromosome, short-read genome sequencing reads were mapped to the final contigs using CLC genomic workbench (read mapping parameters: mismatch cost, 2; insertion cost, 3; deletion cost, 3; length fraction, 0.9; similarity fraction, 0.9; global alignment, no; nonspecific match handling, map randomly; output mode, create stand-alone read mappings; **Supplementary Table 2**).

***RNA extraction from Streptomyces and M. xanthus***

For *Streptomyces*, the cells harvested from the culture plate were resuspended in 3 mL lysis buffer (20 mM Tris-HCl [pH 7.4], 140 mM NaCl, 5 mM MgCl_2_, and 1% Triton X-100) then dropped into a mortar filled with liquid nitrogen and ground using a pestle. The ground cells were thawed and centrifuged at 3,000 × *g* for 10 min at 4°C for removal of cell debris. The supernatants were further cleaned by centrifugation at 16,000 × *g* for 10 min at 4°C. Total RNA was isolated by phenol-chloroform extraction and ethanol precipitation. For *M. xanthus*, cells were resuispended in 3 mL liquied CTT medium and centrifuged at 3,000 × *g* for 15 min at 4°C. Total RNA was isolated from the cell pellet using 1 mL TRIzol (Invitrogen, Carlsbad, CA, USA). Total RNA samples were incubated at 37°C for 1 h with DNase I (NEB, Ipswich, MA, USA) to remove genomic DNA and were further purified by phenol-chloroform extraction and ethanol precipitation. The quality of the isolated total RNA was checked by visualization using 2% agarose gel electrophoresis. rRNA was specifically removed using a Ribo-Zero kit (Epicentre, Madison, WI, USA).

***Quantitative reverse transcription PCR (qPT-PCR)***

cDNA was synthesized using a SuperScript III First-Strand Synthesis System (Invitrogen, Carlsbad, CA, USA) from total RNA samples used for RNA-Seq library construction for RNA-Seq validation and total RNA samples of SCO6666 overexpression strain, repectively. PCR was carried out using Phusion High-Fidelity DNA polymerase (Thermo Fisher Scientific Inc.) and SYBR Green (Invitrogen). The PCR mixtures were cycled at 98°C for 30 s (one cycle), followed by 34 cycles at 98°C for 10 s, 61.6°C for 30 s, and 72°C for 20 s. The amplification profile was monitored on a CFX96 Real-Time PCR Detection System (Bio-Rad, Hercules, CA, USA). Primers used in this study are listed in **Supplementary Table 4**.

***Measurement of iron concentration***

For measuring extracellular iron levels, cells were removed from the culture plate, and 200 mg solid agar medium was sampled and melted in 500 μL QG buffer (Qiagen, Hilden, Germany). To measure intracellular iron levels, *S. coelicolor* and *M. xanthus* were collected from the culture plate (width = 1.5 cm, length = 6 cm) and suspended in 1 mL distilled water. For normalizing iron concentrations between samples, wet cell weight was measured for *S. coelicolor*, whereas cell density (OD_600nm_) was measured for *M. xanthus*. Cells were sonicated at 4°C using a Branson sonifier SFX 550 (Branson Ultrasonics Corp., Danbury, CT, USA; 25% duty, 20 s purse, 40 s rest, total time: 3 min 20 s). Iron levels of prepared samples were measured using an Iron Bio kit for Cedex Bio (Roche Diagnostics, Indianapolis, IN, USA) [3]. The QG buffer and distilled water did not affect the iron level of samples because their iron levels were below the detection limit of the instrument (< 1 μM).

***LC-electrospray ionization (ESI)-MS/MS analysis of myxochelin A***

For myxochelin A extraction, two volumes of methanol were added to the cocultured *M. xanthus* and solid agar media obtained from the contact area (width = 1.5 cm, length = 6 cm) in the culture plates. Samples were incubated at 25°C overnight and supernatant was air-dried and resuspended in 1 mL methanol, which was then analyzed using a Triple Quad 3500 (SCIEX, Framingham, MA, USA) equipped with an ESI source and a Nexera X2 UHPLC system (Shimadzu, Japan). For the UHPLC system, samples were loaded and separated on a Synergi Fusion-RP column (4 μm, 50 × 2 mm; Phenomenex, Torrance, CA, USA) by injecting 5 μL of sample. The column was maintained at 40°C, and the gradient condition started at 5% (v/v) acetonitrile (ACN)/water with 0.1% formic acid and was held for 2 min at a flow rate of 300 μL/min. The ACN content was linearly ramped up to 95% for 6 min. The effluent was determined by positive ion ESI and ion trap MS/MS. Quantification was based on the most abundant product ions from fragmentation of the protonated ion for myxochelin A (*m/z* 405.057 > 268.9).

***Disruption of SCO6666 in S. coelicolor using the CRISPR/Cas9 system***

For gene disruption, a dual-guide RNA cassette was designed and cloned into the *BbsI* site of the pCRISPOmyces-2 vector following the established protocol [4]. Additionally, 1-kb left and right homology arms for SCO6666 deletion were PCR amplified to have 30 bp overlaps at the junction of the two arms and an *XbaI* site at the opposite ends of the two arms. The two homology arms were linked by overlapping extension PCR, and was cloned into the pCRISPomyces-2 vector. The constructed vector was transformed into the methylation-deficient *E. coli* strain ET12567/pUZ8002 [5] and introduced into *S. coelicolor* through intergeneric conjugation [6]. After conjugation, individual colonies were picked and grown in liquid CTT medium at 30°C for 3 days, and the gDNA samples were isolated using a Wizard Genomic DNA Purification Kit (Promega). Gene disruption was confirmed by PCR (**Supplementary Table 4**). Clearance of the introduced vector was accomplished by growing at 37°C for 3 days, followed by replica plating on selective (with apramycin) and nonselective plate (without apramycin). For phenotypic assays, the deletion strain was cocultured with *M. xanthus* as described above.

***Overexpression of SCO6666 in S. coelicolor***

The SCO6666 gene was PCR amplified using LA-taq DNA polymerase (Takara, Japan) with primers containing *XbaI* and *EcoRI* cut sites. The amplified fragment was cloned downstream of the *ermE* promoter in the piBR25 vector. The 400-bp upstream region from the transcription start sites of the two selected genes was PCR amplified with primers containing *SacI* and *XbaI* (NEB) cut sites. The *ermE* promoter located between *SacI* and *XbaI* sites of the piBR25 vector was replaced with the amplified promoter of the two selected genes (**Supplementary Table 4**). The constructed vectors were demethylated in *E. coli* JM110 strain and introduced into *S. coelicolor* using a standard transformation procedure [6].


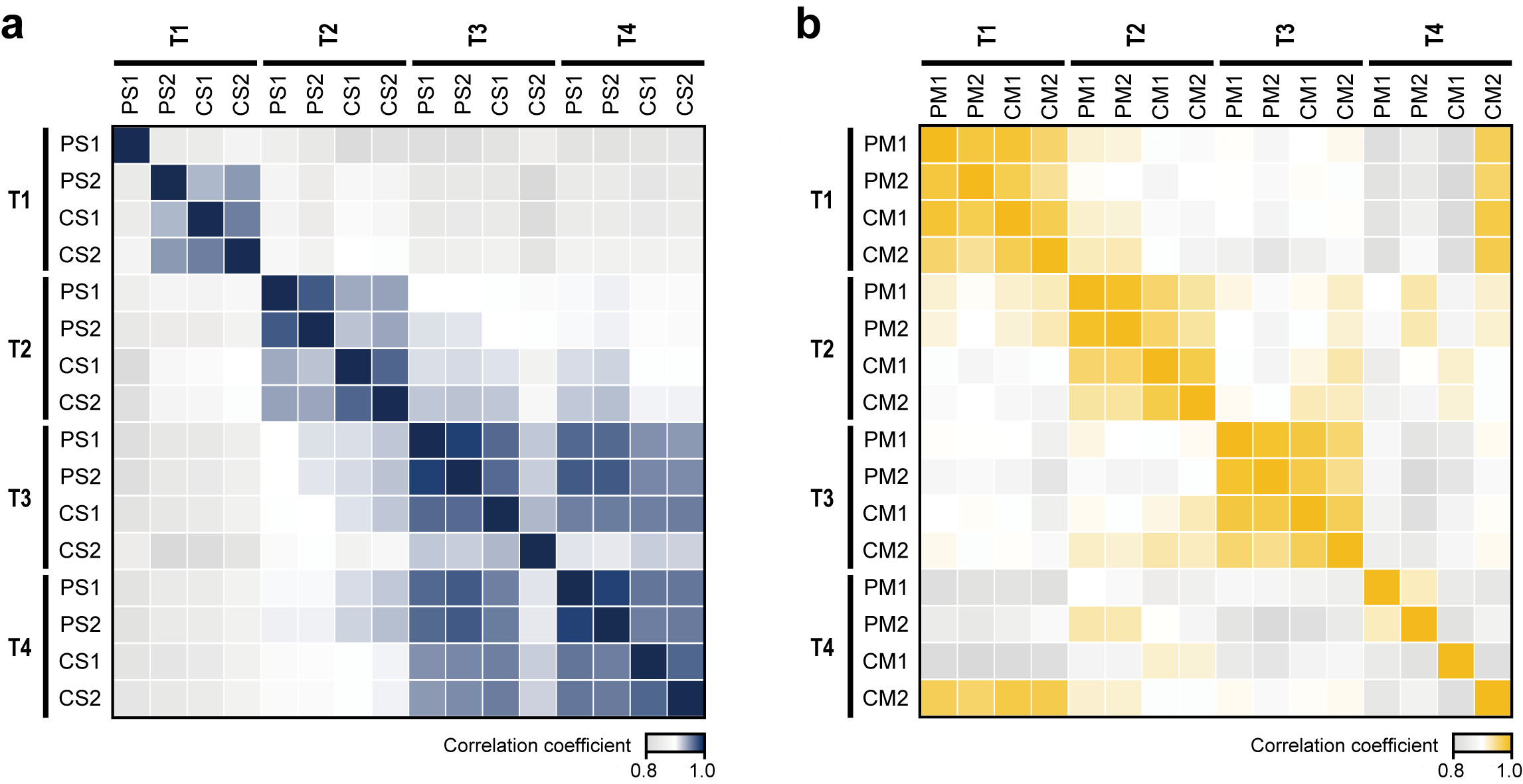


**Supplementary Fig. 1. Reproducibility of co-culture RNA-Seq results.** A total of 14 million sequencing reads on average were obtained from each library. After trimming adaptor sequences and removing reads with low quality (Phred quality score cut off = 0.05), in on average 85.5% and 87.7% of the sequencing reads were mapped to the reference genome sequences of *S. coelicolor* (NC_003888) and *M. xanthus* (NC_008095), respectively (Supplementary Table S1). After normalization using the DESeq2 package in R [7], the calculation of pairwise correlation coefficients (> 0.8) demonstrated a high reproducibility between the biological replicates. Gene expression in the co-cultured samples was similar to that in the pure-cultured samples at the same time points, indicating that the culture time (i.e., growth phase) is the main factor affecting gene expression. **(a)** Reproducibility of *S. coelicolor* RNA-seq results. The RNA-Seq results were highly reproducible (*R* > 0.8). In the label, the number followed by the sample name indicates a duplicate at same time point. **T1**, time point 1; **T2**, time point 2; **T3**, time point 3; **T4**, time point 4; **CS**, co-cultured *S. coelicolor*; **PS**, pure-cultured *S. coelicolor*. **(b)** Reproducibility of *M. xanthus* RNA-seq results. The RNA-Seq results were highly reproducible (*R* > 0.8). **CM**, co-cultured *M. xanthus*; **PM**, pure-cultured *M. xanthus*.


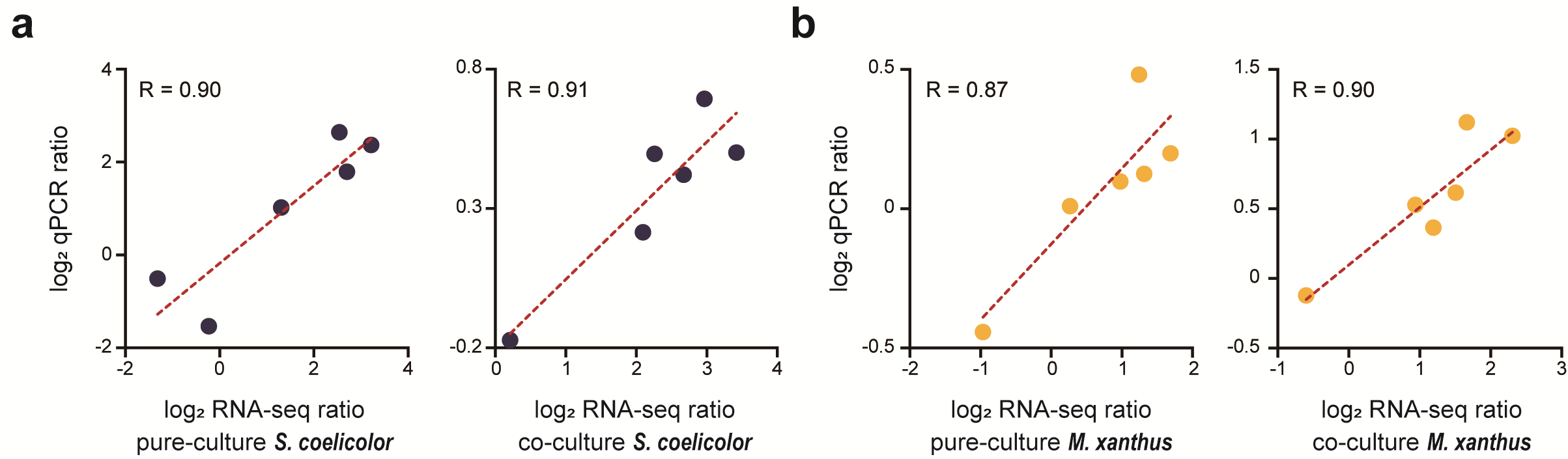


**Supplementary Fig. 2. Validation of RNA-Seq results using quantitative reverse transcription PCR (qRT-PCR).** RNA-Seq and quantitative reverse transcription PCR (qRT-PCR) expression values were highly correlated (R > 0.87), confirming the accuracy of RNA-Seq results. qRT-PCR method is fully described in **Supplementary Methods**. **(a)** Validation of *S. coelicolor* RNA-Seq data by comparison between RNA-Seq and qRT-PCR. qRT-PCR was performed using RNA samples which were used to construct T2 RNA-Seq library. The expression ratio from qRT-PCR was calculated from the Ct value difference between target gene and *SCO1922* which has constant RNA-Seq reads at T2 of pure-culture and co-culture condition. RNA-Seq ratio was calculated by dividing RNA-Seq reads mapped to target gene by RNA-Seq reads mapped to *SCO1922*. **(b)** Validation of *M. xanthus* RNA-Seq data by comparison between RNA-Seq and qRT-PCR. The expression ratio from qRT-PCR was calculated from the Ct value difference between target gene and *MXAN_5877* which has constant RNA-Seq reads at T2 of pure-culture and co-culture condition.


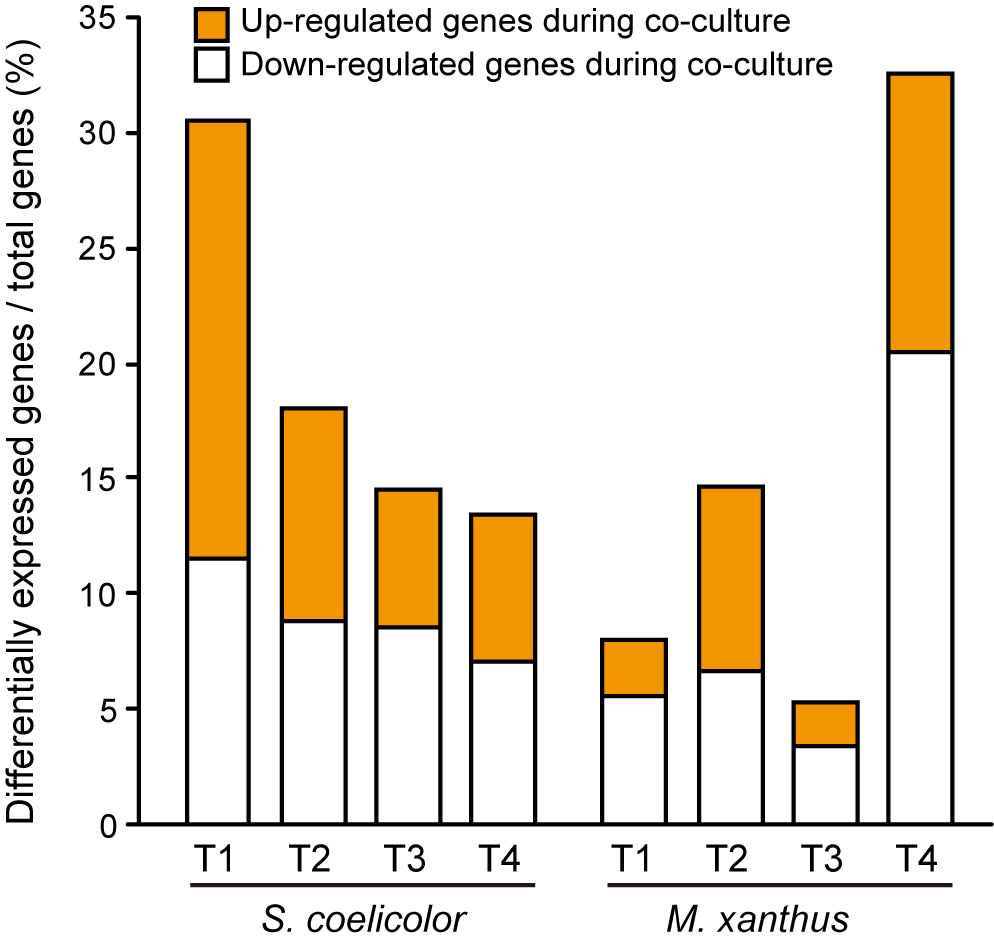


**Supplementary Fig. 3. The proportion of the differentially expressed genes**. To determine the genes whose transcription is influenced by the co-culture condition, fold changes of each gene were calculated by dividing the normalized gene expression value of co-culture by that of pure-culture. Among 7,767 protein coding genes of *S. coelicolor*, about 19% of genes were identified as differentially expressed genes (DEGs) between the pure- and the co-culture conditions at each of the four time points (P-value < 0.05). In the case of *M. xanthus*, among 7,247 genes, on average 16% of genes were determined as DEGs during co-culture relative to pure-culture. Orange box represents proportion of up-regulated genes during co-culture at each time point. White box indicates proportion of down-regulated genes during co-culture at each time point. **T1**, time point 1; **T2**, time point 2; **T3**, time point 3; **T4**, time point 4.


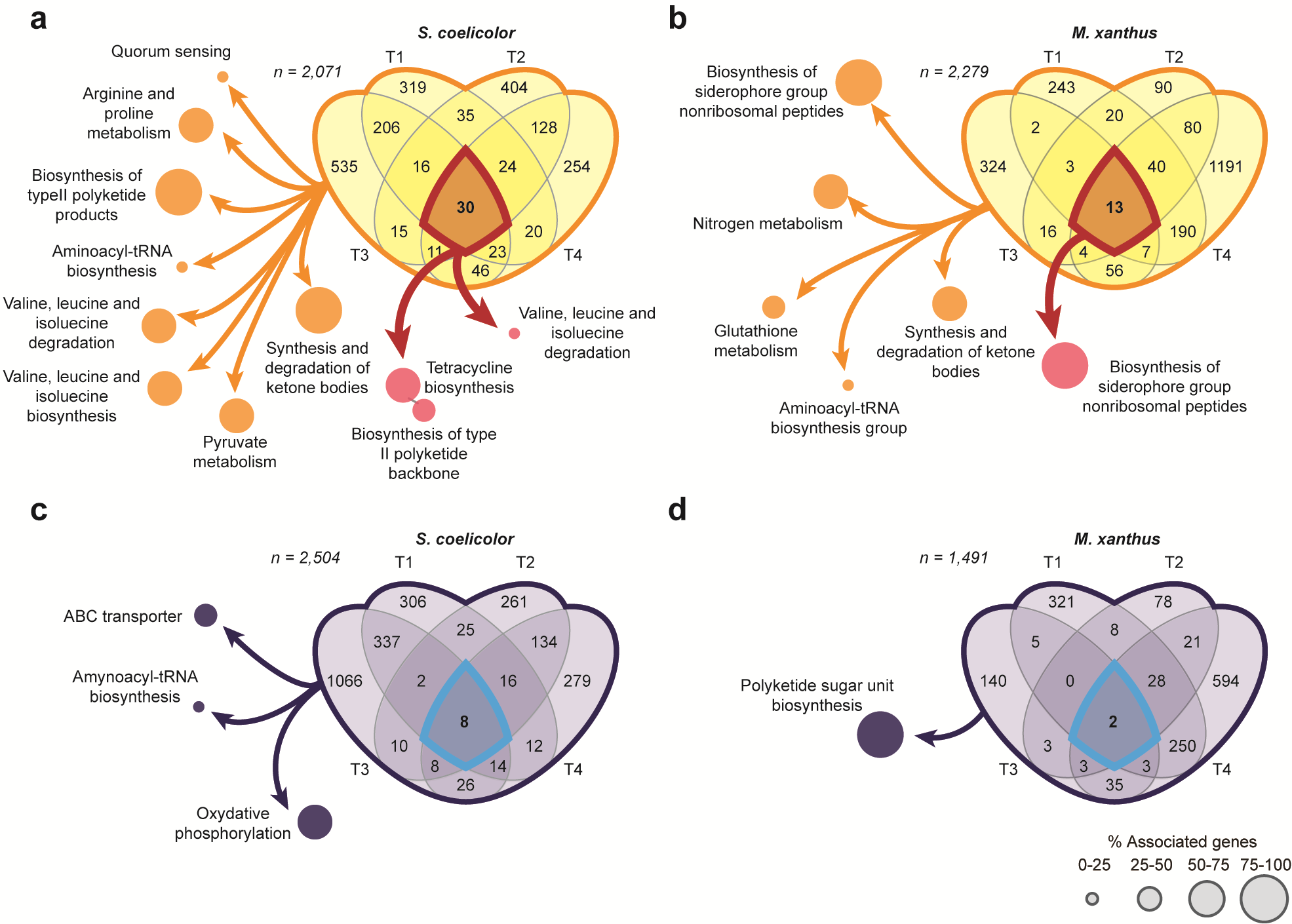


**Supplementary Fig. 4. Pathway enrichment analysis of DEGs during the co-culture.** Size of circle indicates percent of term associated genes. *p*-value < 0.05 was considered as the cut off criteria for each enriched term. **T1**, time point 1; **T2**, time point 2; **T3**, time point 3; **T4**, time point 4. **(a)** Enriched KEGG pathway among the up-regulated genes of *S. coelicolor* during co-culture. Orange closed circles represent pathway, enriched among total significantly up-regulated genes in co-culture condition compared to pure-culture condition at any one time point. Red closed circles indicate pathway, enriched among statistically significantly up-regulated genes at all four time points. **(b)** Enriched KEGG pathway among the up-regulated genes of *M. xanthus* during co-culture. **(c)** Enriched KEGG pathway among the down-regulated genes of *S. coelicolor* during co-culture. **(d)** Enriched KEGG pathway among the down-regulated genes of *M. xanthus* during co-culture.

**
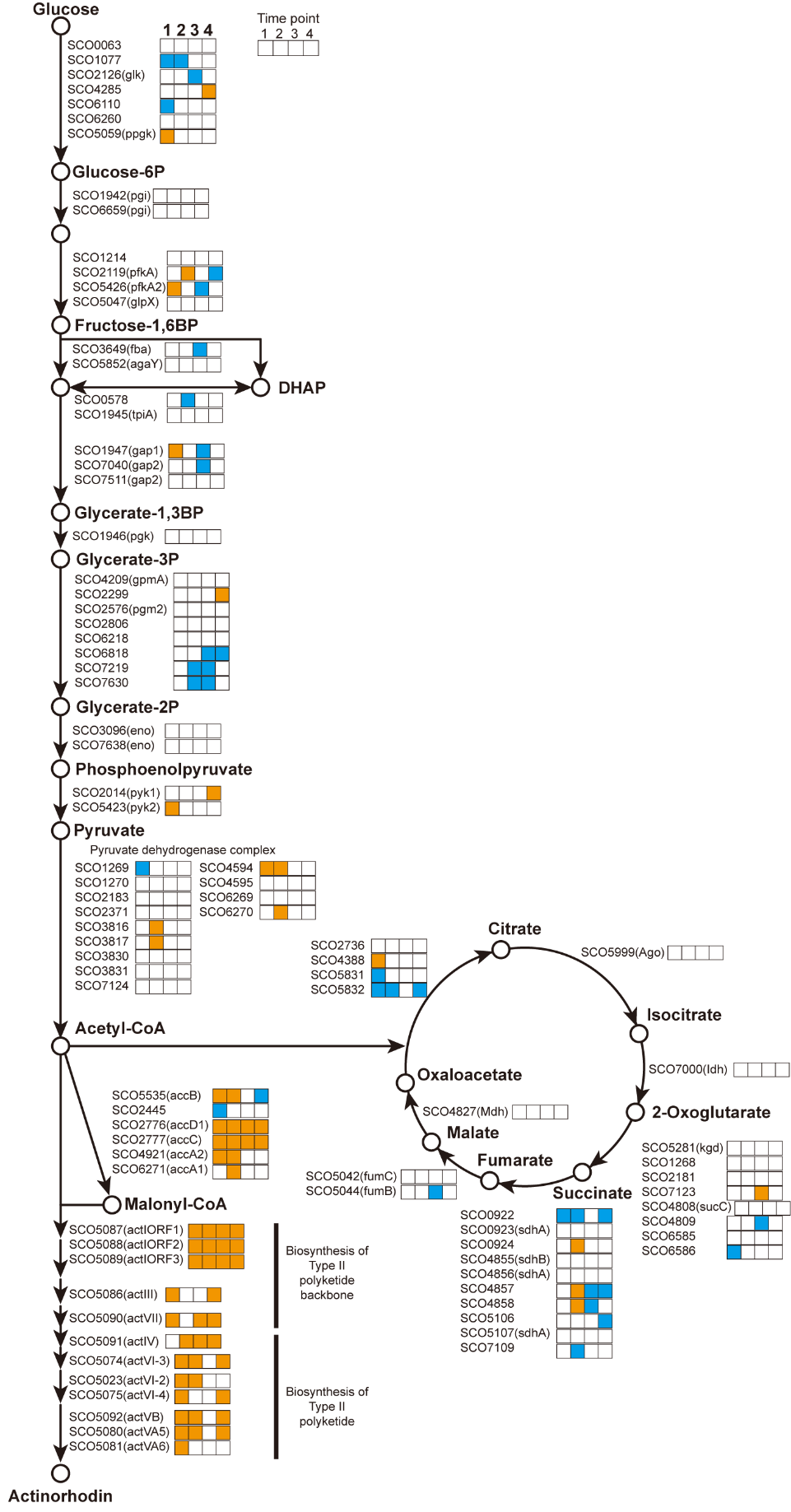
**

**Supplementary Fig. 5. Transcriptomic profile of primary metabolism and actinorhodin biosynthesis of *S. coelicolor*.** The four boxes next to the gene name denote time point 1 through 4 in order. The orange boxes indicate genes statistically significantly (*p*-value < 0.05) up-regulated and skyblue boxes indicates the gene statistically significantly down-regulated (*p*-value < 0.05) at the corresponding time point.


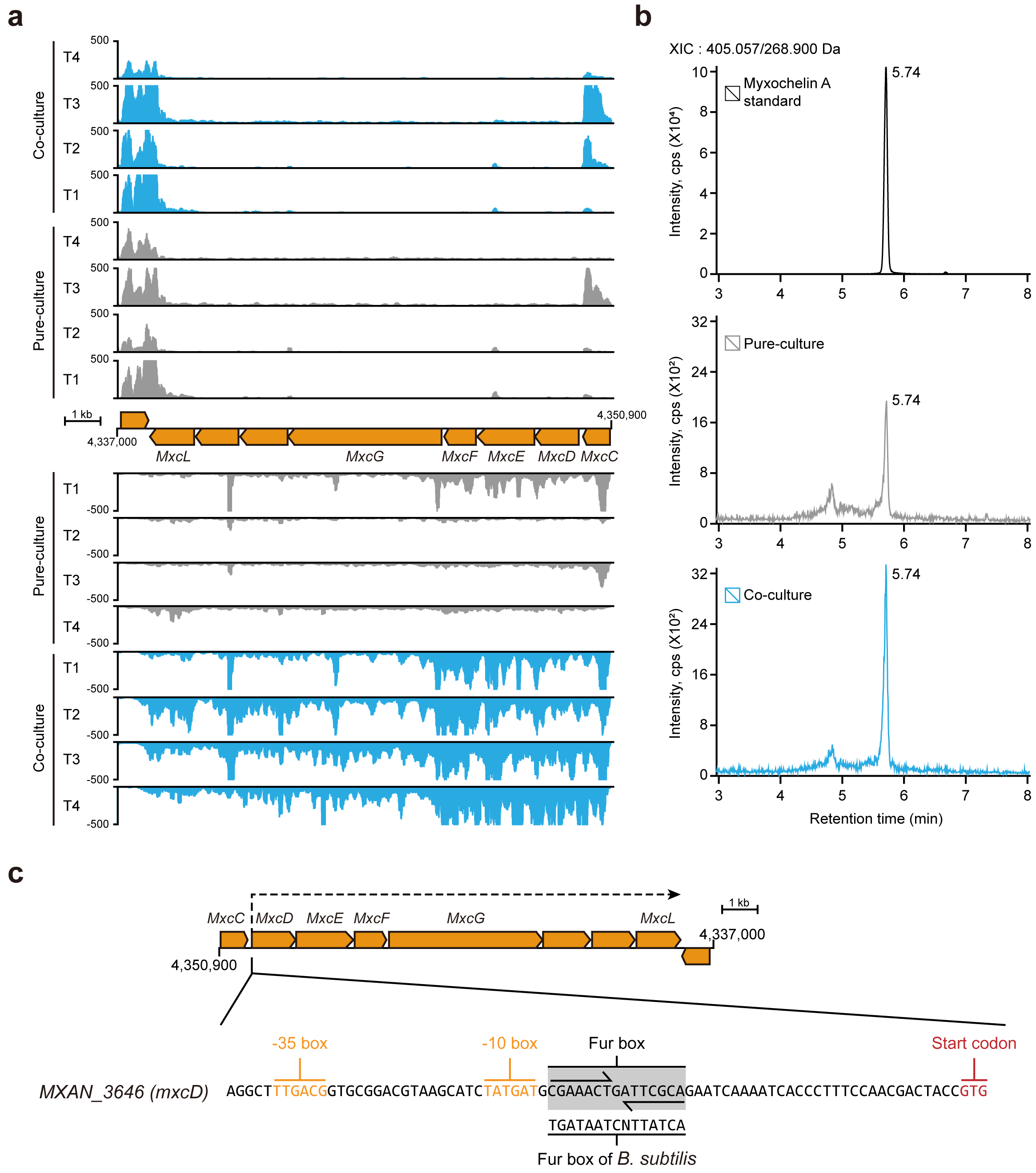


**Supplementary Fig. 6. Myxochelin production of *M. xanthus* during co-culture. (a)** Differential expression pattern of myxochelin biosynthetic cluster of *M. xanthus* at pure-culture and co-culture condition. Numbers below the start and end position of the cluster indicate the position in the genome. **T1**, time point 1; **T2**, time point 2; **T3**, time point 3; **T4**, time point 4. **(b)** Myxochelin production of *M. xanthus* during pure-culture and co-culture. Numbers above the peak is exact retention time. **(c)** Putative binding site of Fur in myxochelin biosynthetic cluster. Dashed arrow indicates the operon structure predicted by DOOR database.


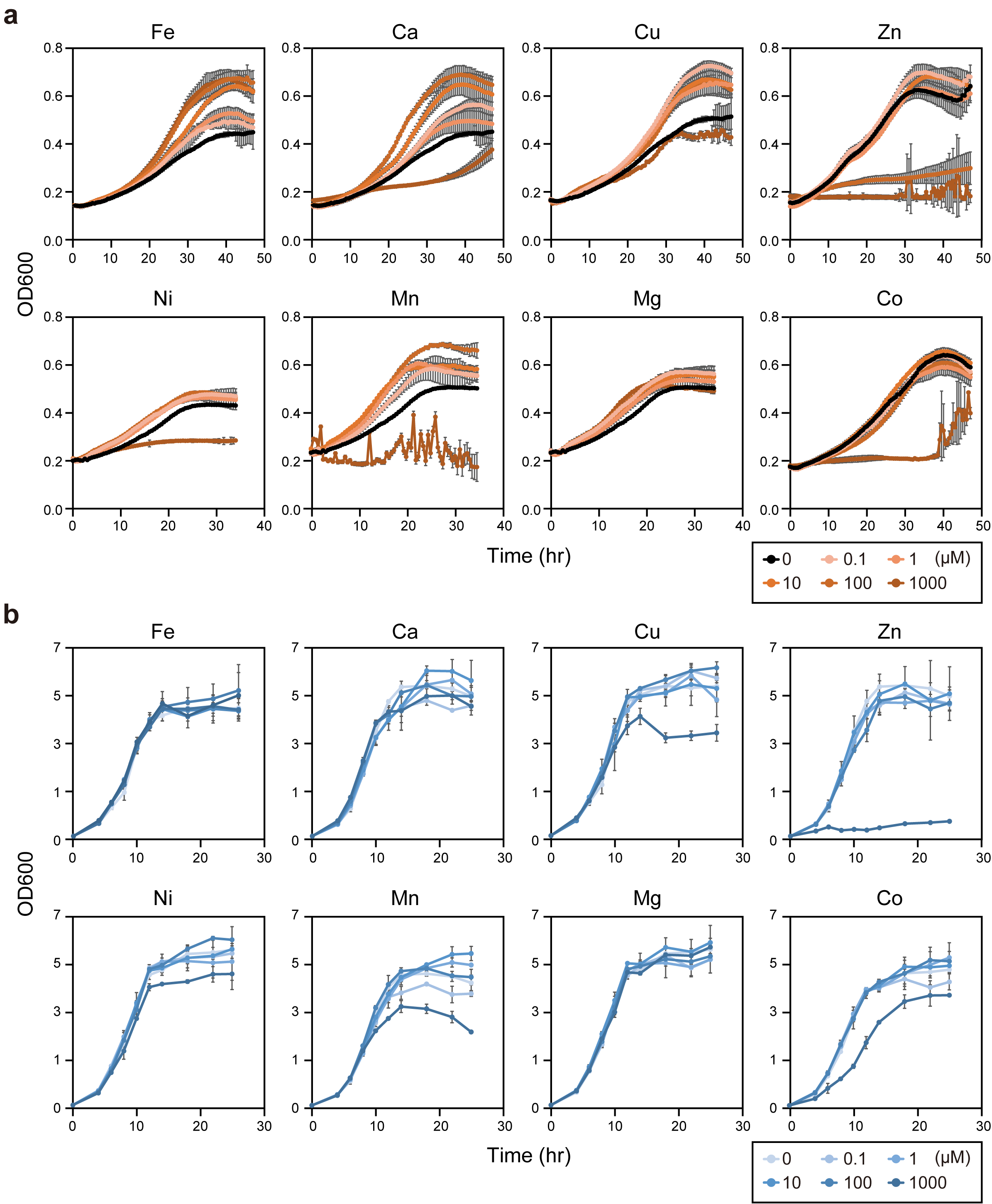


**Supplementary Fig. 7. Growth profile under various metal treatment. (a)** Growth profile of *M. xanthus* at CTT liquid media under various metal treatment. **(b)** Growth profile of *S. coelicolor* at CTT liquid media under various metal treatment.


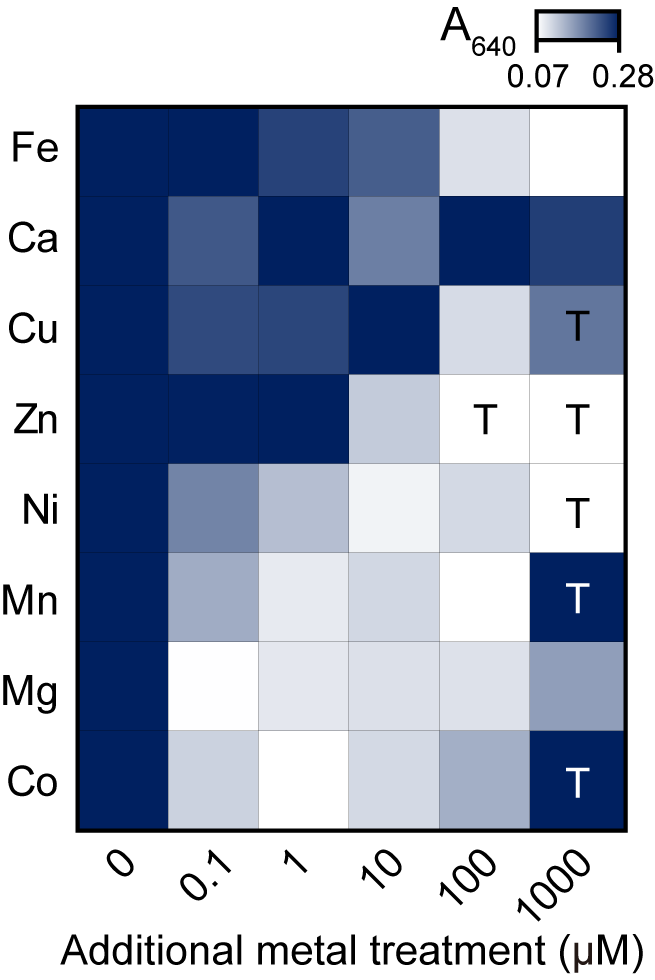


**Supplementary Fig. 8. Actinorhodin production of *S. coelicolor* during co-culture with *M. xanthus* under additional metal treatment.** Large T on the box indicates that growth retardation was observed on *S. coelicolor* or *M. xanthus* at corresponding metal treatment condition.


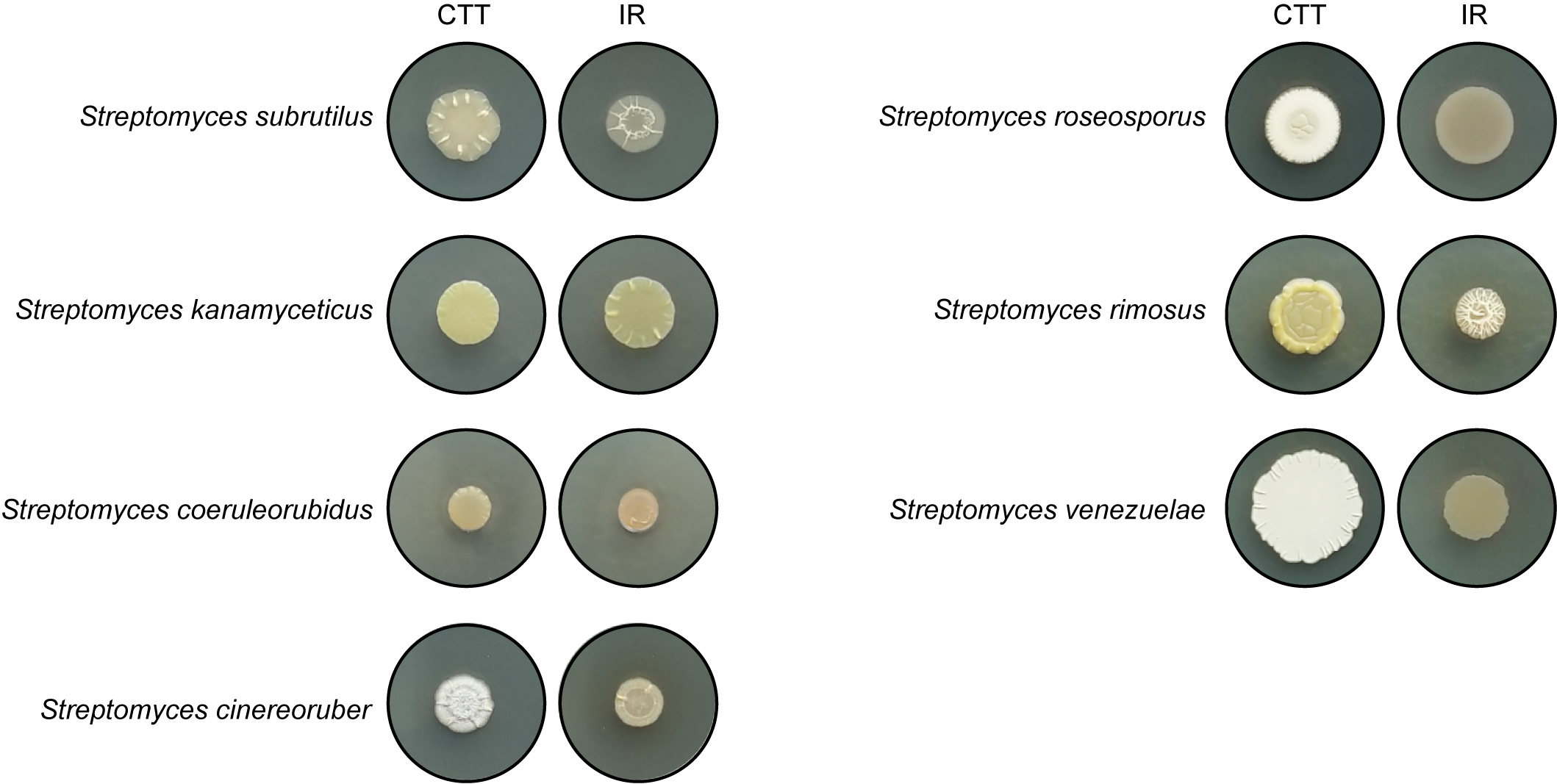


**Supplementary Fig. 9. Colony morphology of seven *Streptomyces* species in the iron restricted condition. CTT**, pure-cultured in CTT solid media; **IR**, pure-cultured in iron restricted CTT solid media (250 μM 2,2’-bipyridyl).


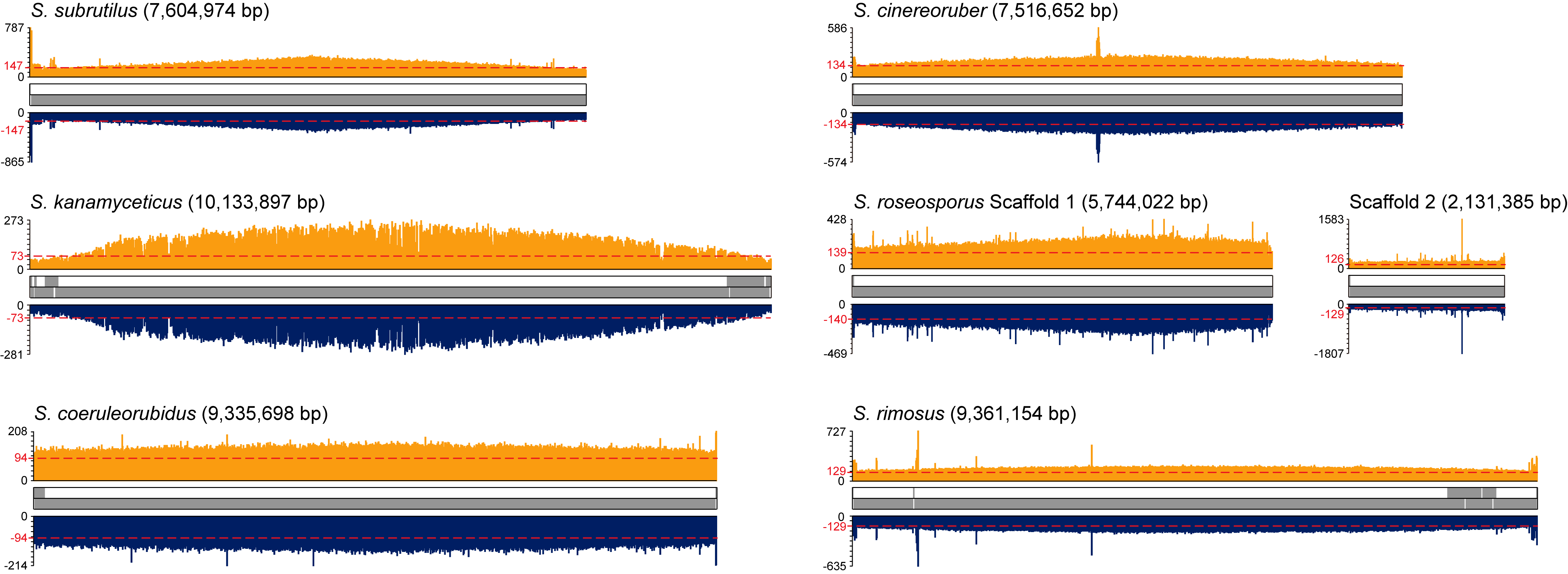


**Supplementary Fig. 10. Genome completion of six *Streptomyces* species.** Each gray box indicates assembled contigs from the short-read genome sequencing or long-read genome sequencing. Peaks represent the number of mapped short-read genome sequencing reads on the corresponding genomic position. Each red dashed line indicates average of mapped read number on the whole genomic positions.


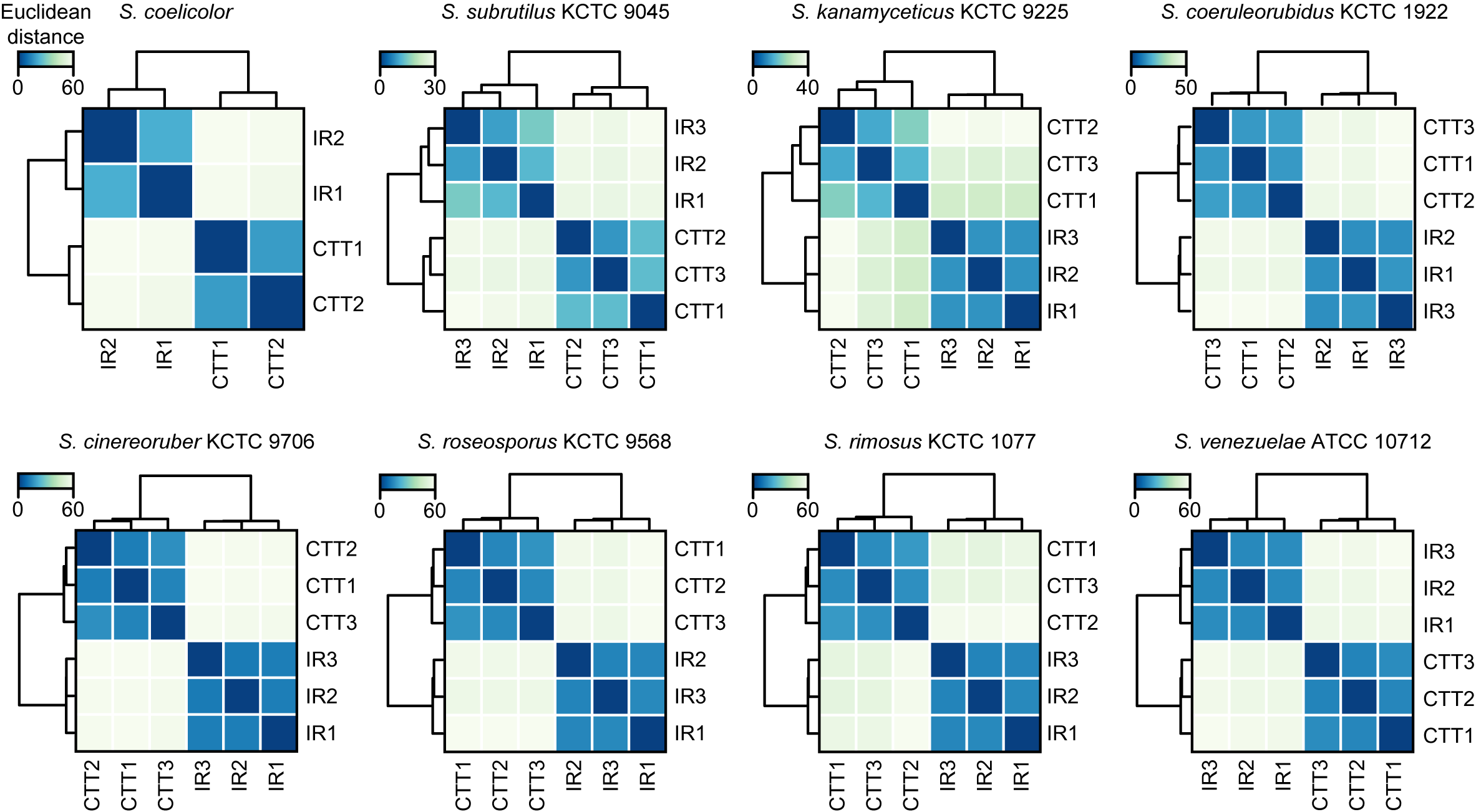


**Supplementary Fig. 11. Reproducibility of iron restricted condition RNA-Seq results.** A total of 22 million sequencing reads on average were obtained from each library. After trimming adaptor sequences and removing reads with low quality (Phred quality score cut off = 0.05), in on average 96.3% of the sequencing reads were mapped to the reference genome sequences (**Supplementary Table 3**). Reproducibility of RNA-Seq results was validated by calculation of Euclidean distance between samples by using the DESeq2 package in R[7]. In the label, the number followed by the sample name indicates a biological replicates. **CTT**, pure-cultured in CTT solid media; **IR**, pure-cultured in iron restricted CTT solid media (250 μM 2,2’-bipyridyl).


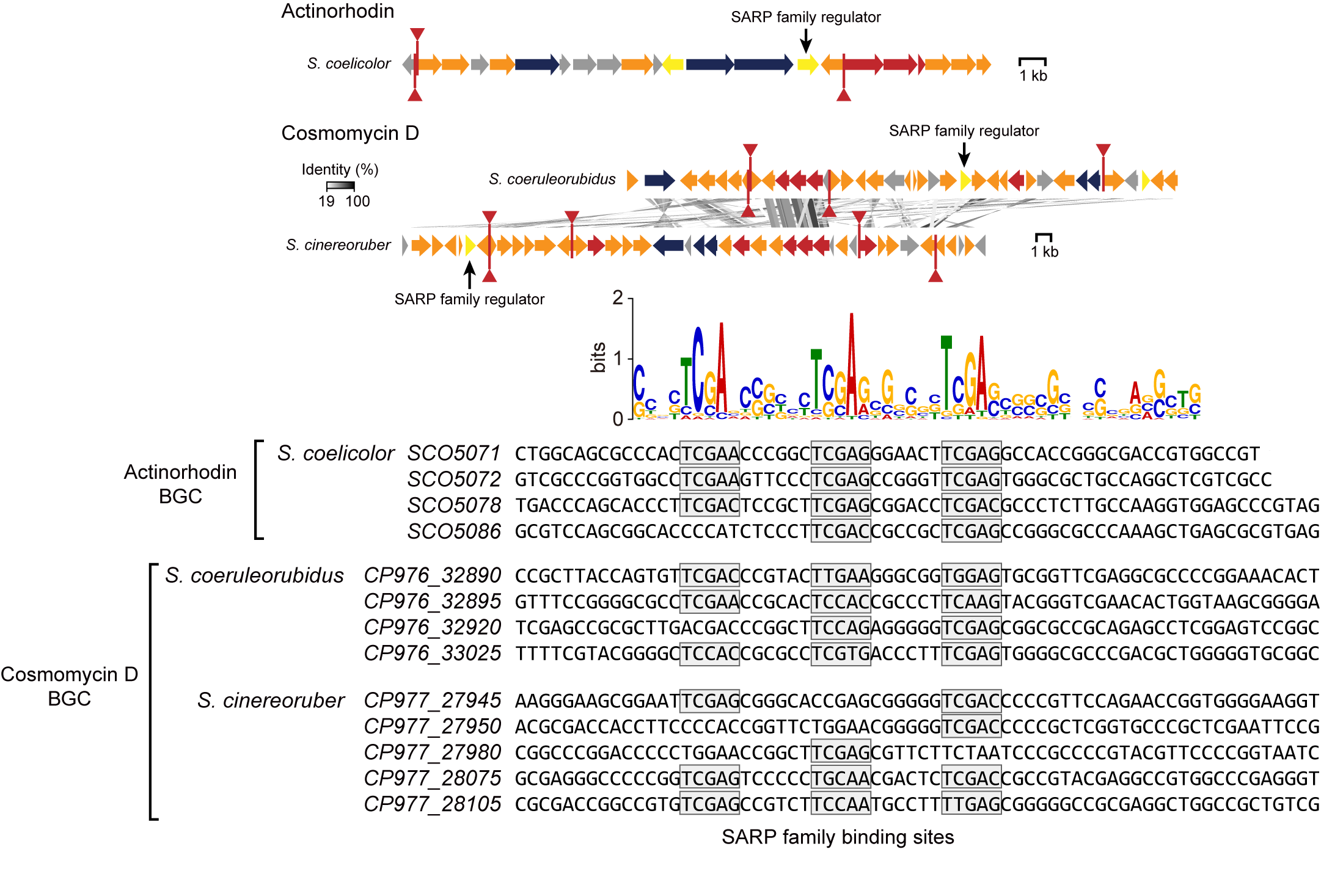


**Supplementary Fig. 12. Analysis of promoter region in the six non-siderophore BGCs up-regulated in the iron restricted condition.** Operon structure of six non-siderophore BGCs were predicted using Operon-mapper, web based program[8]. Considering the operon structure, motif search was performed on the all 200 bp upstream region from the start codon of the first gene in each operon encoded in six non-siderophore BGCs. Only one conserved motif was searched in the four promoter regions in actinorhodin BGC and nine promoter regions in cosmomycin BGC (E-value < 10^-9^). This motif resembles the binding sites of SARP family binding sites[9].

**
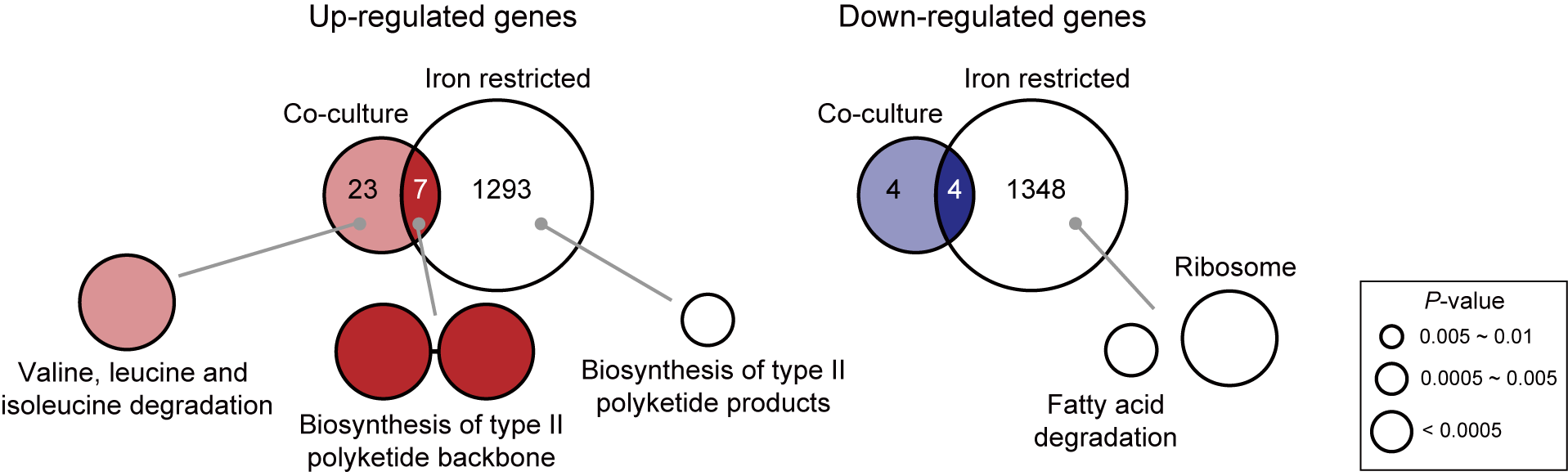
**

**Supplementary Fig. 13. Differentially expressed genes and enriched KEGG pathway of *S. coelicolor* during co-culture and iron restricted condition.** The enriched biological function categories of DEGs was screened by using ClueGO using KEGG pathway terms (*P*-value < 0.01)[10].


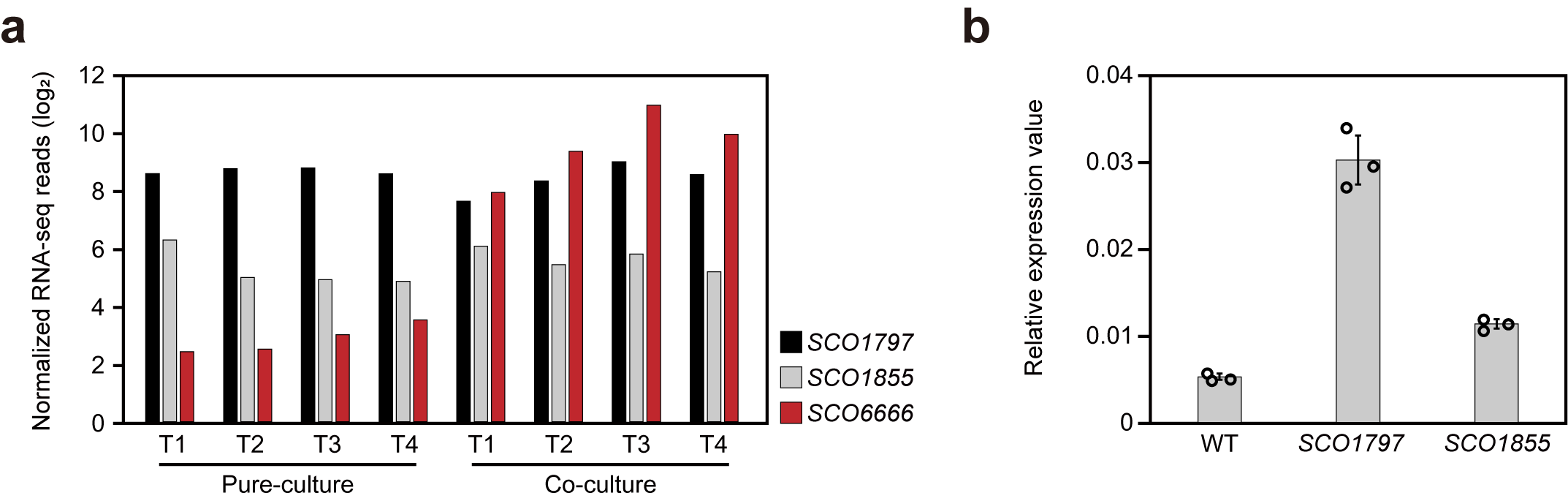


**Supplementary Fig. 14. SCO6666 overexpression in *S. coelicolor*.** (a) RNA expression level of SCO6666 and selected six genes in pure-culture and co-culture condition throughout the growth. **T1**, time point 1; **T2**, time point 2; **T3**, time point 3; **T4**, time point 4. (b) Validation of SCO6666 overexpression strains using qRT-PCR. qRT-PCR was performed using RNA samples which were extracted from each strains cultured on CTT solid media. Detailed method for qRT-PCR is described in **Supplementary Methods**. The expression ratio from qPCR was calculated from the Ct value difference between SCO6666 and *hrdB* (SCO5820) which is well known house keeping gene of *S. coelicolor*. **WT**, wild type *S. coelicolor*; **SCO1797**, strain with SCO1797 promoter; **SCO1855**, strain with SCO1855 promoter.

**Supplementary Table 1. Genome completion of six *Streptomyces* species.**

| Species | Number of assembled scaffold | Genome length  (bp) | Illumina total read | Illumina mapped read | Illumina mapped read percentage (%) |
| --- | --- | --- | --- | --- | --- |
| *Streptomyces subrutilus* | 1 | 7,604,974 | 23,157,337 | 22,315,389 | 96.36 |
| *Streptomyces kanamyceticus* | 1 | 10,133,897 | 14,877,044 | 14,723,577 | 98.97 |
| *Streptomyces coeruleorubidus* | 1 | 9,335,698 | 17,546,866 | 17,478,403 | 99.61 |
| *Streptomyces cinereoruber* | 1 | 7,516,652 | 20,181,890 | 20,123,294 | 99.71 |
| *Streptomyces roseosporus* | 2 | 5,744,022 / 2,131,385 | 21,180,615 | 15932688 / 5143847 | 99.51 |
| *Stretpomyces rimosus* | 1 | 9,361,154 | 25,108,764 | 24,148,183 | 96.17 |

**Supplementary Table 2. Co-culture RNA-Seq read mapping statistics.** A total of 14 million sequencing reads on average were obtained from each library. After trimming adaptor sequences and removing reads with low quality (Phred quality score cut off = 0.05), sequencing reads were mapped to the reference genome sequences of *S. coelicolor* (NC_003888) and *M. xanthus* (NC_008095) using CLC Genomics Workbench software (CLC Bio, Aarhus, Denmark) with the following parameters: mismatch costs, 2; length fraction, 0.9; and similarity fraction, 0.9.

| Sample | Total reads | Mapped reads | Mapping percentage (%) |
| --- | --- | --- | --- |
| T1_PS1 | 14606840 | 12979646 | 88.86 |
| T1_PS2 | 11008211 | 8694044 | 78.98 |
| T1_CS1 | 12380107 | 10707479 | 86.49 |
| T1_CS2 | 14331386 | 11481092 | 80.11 |
| T1_PM1 | 13118644 | 11656957 | 88.86 |
| T1_PM2 | 14241632 | 13044670 | 91.60 |
| T1_CM1 | 13516665 | 12032694 | 89.02 |
| T1_CM2 | 14246091 | 13309366 | 93.42 |
| T2_PS1 | 14695387 | 11884482 | 80.87 |
| T2_PS2 | 14083870 | 12887422 | 91.50 |
| T2_CS1 | 13349602 | 10120453 | 75.81 |
| T2_CS2 | 12080847 | 8036106 | 66.52 |
| T2_PM1 | 14701237 | 12397637 | 84.33 |
| T2_PM2 | 12994345 | 11622317 | 89.44 |
| T2_CM1 | 14292522 | 12822227 | 89.71 |
| T2_CM2 | 13729763 | 11097188 | 80.83 |
| T3_PS1 | 14828796 | 13801314 | 93.07 |
| T3_PS2 | 14103463 | 13578581 | 96.28 |
| T3_CS1 | 13161515 | 11431760 | 86.86 |
| T3_CS2 | 12054209 | 10412261 | 86.38 |
| T3_PM1 | 14073400 | 13003695 | 92.40 |
| T3_PM2 | 14503253 | 13510896 | 93.16 |
| T3_CM1 | 17017468 | 16011256 | 94.09 |
| T3_CM2 | 14710292 | 13486561 | 91.68 |
| T4_PS1 | 12930779 | 12511519 | 96.76 |
| T4_PS2 | 14485536 | 13467805 | 92.97 |
| T4_CS1 | 19192274 | 16887549 | 87.99 |
| T4_CS2 | 12582185 | 11223603 | 89.20 |
| T4_PM1 | 13221247 | 10998177 | 83.19 |
| T4_PM2 | 12451904 | 9884670 | 79.38 |
| T4_CM1 | 14695199 | 12186836 | 82.93 |
| T4_CM2 | 12964319 | 10358779 | 79.90 |

**Supplementary Table 3. Iron restricted condition RNA-Seq read mapping statistics.** A total of 22 million sequencing reads on average were obtained from each library. After trimming adaptor sequences and removing reads with low quality (Phred quality score cut off = 0.05), sequencing reads were mapped to the completed genome sequence of each species using CLC Genomics Workbench software (CLC Bio, Aarhus, Denmark) with the following parameters: mismatch costs, 2; length fraction, 0.9; and similarity fraction, 0.9.

| Sample | Condition | Species | Total reads | Mapped reads | Mapped read percent (%) |
| --- | --- | --- | --- | --- | --- |
| Sco_CTT1 | Normal CTT media | *Streptomyces coelicolor* | 5096859 | 4890427 | 95.95 |
| Sco_CTT2 | Normal CTT media | *Streptomyces coelicolor* | 5064205 | 4864076 | 96.05 |
| Sco_IR1 | iron-restriced CTT media | *Streptomyces coelicolor* | 3091146 | 2943786 | 95.23 |
| Sco_IR2 | iron-restriced CTT media | *Streptomyces coelicolor* | 3761043 | 3557746 | 94.59 |
| Sco_R5(-)_solid1 | Normal R5(-) media | *Streptomyces coelicolor* | 10120777 | 8715368 | 86.11 |
| Sco_R5(-)_solid2 | Normal R5(-) media | *Streptomyces coelicolor* | 12478171 | 10362637 | 83.05 |
| Ssub_CTT1 | Normal CTT media | *Streptomyces subrutilus* | 23160914 | 22314854 | 96.35 |
| Ssub_CTT2 | Normal CTT media | *Streptomyces subrutilus* | 24292117 | 23218987 | 95.58 |
| Ssub_CTT3 | Normal CTT media | *Streptomyces subrutilus* | 26282526 | 25342449 | 96.42 |
| Ssub_IR1 | iron-restriced CTT media | *Streptomyces subrutilus* | 26693850 | 25707315 | 96.30 |
| Ssub_IR2 | iron-restriced CTT media | *Streptomyces subrutilus* | 23705397 | 22906641 | 96.63 |
| Ssub_IR3 | iron-restriced CTT media | *Streptomyces subrutilus* | 23051738 | 22041370 | 95.62 |
| Skan_CTT1 | Normal CTT media | *Streptomyces kanamyceticus* | 22053729 | 21124330 | 95.79 |
| Skan_CTT2 | Normal CTT media | *Streptomyces kanamyceticus* | 23985468 | 22739288 | 94.80 |
| Skan_CTT3 | Normal CTT media | *Streptomyces kanamyceticus* | 26252807 | 25351781 | 96.57 |
| Skan_IR1 | iron-restriced CTT media | *Streptomyces kanamyceticus* | 25187994 | 24375958 | 96.78 |
| Skan_IR2 | iron-restriced CTT media | *Streptomyces kanamyceticus* | 23628125 | 22945076 | 97.11 |
| Skan_IR3 | iron-restriced CTT media | *Streptomyces kanamyceticus* | 23340839 | 22549340 | 96.61 |
| Scoe_CTT1 | Normal CTT media | *Streptomyces coeruleorubidus* | 22660499 | 22185640 | 97.90 |
| Scoe_CTT2 | Normal CTT media | *Streptomyces coeruleorubidus* | 25081068 | 24635765 | 98.22 |
| Scoe_CTT3 | Normal CTT media | *Streptomyces coeruleorubidus* | 24163492 | 23670283 | 97.96 |
| Scoe_IR1 | iron-restriced CTT media | *Streptomyces coeruleorubidus* | 25876759 | 25445363 | 98.33 |
| Scoe_IR2 | iron-restriced CTT media | *Streptomyces coeruleorubidus* | 24821526 | 24419906 | 98.38 |
| Scoe_IR3 | iron-restriced CTT media | *Streptomyces coeruleorubidus* | 23445921 | 23078628 | 98.43 |
| Scin_CTT1 | Normal CTT media | *Streptomyces cinereoruber* | 24726204 | 24208154 | 97.90 |
| Scin_CTT2 | Normal CTT media | *Streptomyces cinereoruber* | 23270287 | 23007670 | 98.87 |
| Scin_CTT3 | Normal CTT media | *Streptomyces cinereoruber* | 22581104 | 22395901 | 99.18 |
| Scin_IR1 | iron-restriced CTT media | *Streptomyces cinereoruber* | 24260406 | 23681490 | 97.61 |
| Scin_IR2 | iron-restriced CTT media | *Streptomyces cinereoruber* | 24923013 | 24520097 | 98.38 |
| Scin_IR3 | iron-restriced CTT media | *Streptomyces cinereoruber* | 24655924 | 24092569 | 97.72 |
| Sros_CTT1 | Normal CTT media | *Streptomyces roseosporus* | 24903089 | 24156327 | 97.00 |
| Sros_CTT2 | Normal CTT media | *Streptomyces roseosporus* | 26228967 | 25474053 | 97.12 |
| Sros_CTT3 | Normal CTT media | *Streptomyces roseosporus* | 24137621 | 23677455 | 98.09 |
| Sros_IR1 | iron-restriced CTT media | *Streptomyces roseosporus* | 25445779 | 24419461 | 95.97 |
| Sros_IR2 | iron-restriced CTT media | *Streptomyces roseosporus* | 22864537 | 21880046 | 95.69 |
| Sros_IR3 | iron-restriced CTT media | *Streptomyces roseosporus* | 23418785 | 22330716 | 95.35 |
| Srim_CTT1 | Normal CTT media | *Stretpomyces rimosus* | 24005862 | 23068744 | 96.10 |
| Srim_CTT2 | Normal CTT media | *Stretpomyces rimosus* | 24155849 | 23099359 | 95.63 |
| Srim_CTT3 | Normal CTT media | *Stretpomyces rimosus* | 27939220 | 27009577 | 96.67 |
| Srim_IR1 | iron-restriced CTT media | *Stretpomyces rimosus* | 24040830 | 22756771 | 94.66 |
| Srim_IR2 | iron-restriced CTT media | *Stretpomyces rimosus* | 23178910 | 21900140 | 94.48 |
| Srim_IR3 | iron-restriced CTT media | *Stretpomyces rimosus* | 22785385 | 21699770 | 95.24 |
| Sve_CTT1 | Normal CTT media | *Stretpomyces venezuelae* | 25067530 | 24608985 | 98.17 |
| Sve_CTT2 | Normal CTT media | *Stretpomyces venezuelae* | 26614855 | 25992126 | 97.66 |
| Sve_CTT3 | Normal CTT media | *Stretpomyces venezuelae* | 31143890 | 30495365 | 97.92 |
| Sve_IR1 | iron-restriced CTT media | *Stretpomyces venezuelae* | 27335407 | 26748641 | 97.85 |
| Sve_IR2 | iron-restriced CTT media | *Stretpomyces venezuelae* | 25714694 | 24936449 | 96.97 |
| Sve_IR3 | iron-restriced CTT media | *Stretpomyces venezuelae* | 24626923 | 23812090 | 96.69 |

**Supplementary Table 4. Sequence of oligonucleotides used in this study**

| *S. coelicolor* qPCR target | | |
| --- | --- | --- |
| Gene | Forward | Reverse |
| SCO4979 | AGGACCGGACCTTCATCTG | CGCCTTCTCCCTTGTCAC |
| SCO4655 | GCTCGACGTCAACTTCTTCG | GGTGCGGTAGTTGATGGTCT |
| SCO3404 | AACGACAACAGGGTCGAGTC | TGTAGCTCGCCTGGATCTTC |
| SCO5147 | GAGGACCTCACCCAAGAGG | TCGAGGAAGAGATTGGTCGT |
| SCO5737 | CACCATCCGCTTCGAGAC | GTCCTTCGGGTTCTTGGAG |
| SCO3537 | GTTCTGCCACTCGACCTCTC | GATCTTTGTCGCCAACTCCT |
| SCO1922 | TCACCCGAAGTACACGATCA | GTACTGCATGGCGAGGAAC |
| SCO3373 | CACCGAGCACATCCTCCT | ATGATCTCCTCCACCTGCTG |
| SCO2999 | GACTACGCATCCGTGTTCG | CAGCCGGTAGTGCGAGAC |
| *M. xanthus* qPCR target | | |
| Gene | Forward | Reverse |
| *MXAN_1264* | GCATCGTCTGGTGTGACG | AGCGGTGGAGGTAGCTGTT |
| *MXAN_4219* | GAGCTGTTCCACCATGTCC | GCACCCTTGGTGACAATCTT |
| *MXAN_4327* | GGAGATCTTCCATCGTGCAG | AGGCGGTCCTTCAGCTTC |
| *MXAN_3077* | GCGAAGATCATCGACATTCC | CTTGAAGACGCCCTGAAGAC |
| *MXAN_2667* | CTCGCCGAGGAAATGGAAC | GCTCCCGAACTTGTCCAG |
| *MXAN_0886* | TCAGGTGTCGCTGAAATGTT | AATAAACGTGGGGACATTCG |
| *MXAN_5877* | AAGGTGGATGACTCGCACTC | CTTGTCATCCAGGTTCAACG |
| Oligo used in *SCO6666* disruption | | |
| Oligo | Sequence | |
| SCO6666 synthetic dual gRNA cassette | GAGACATCTTTGAAGACAAACGCACGTCACGAAAGCGCCGGGTGTTTTAGAGCTAGAAATAGCAAGTTAAAATAAGGCTAGTCCGTTATCAACTTGAAAAAGTGGCACCGAGTCGGTGCTTTTTTAGCATAACCCCTTGGGGCCTCTAAACGGGTCTTGAGGGGTTTTTTGGCTGCTCCTTCGGTCGGACGTGCGTCTACGGGCACCTTACCGCAGCCGTCGGCTGTGCGACACGGACGGATCGGGCGAACTGGCCGATGCTGGGAGAAGCGCGCTGCTGTACGGCGCGCACCGGGTGCGGAGCCCCTCGGCGAGCGGTGTGAAACTTCTGTGAATGGCCTGTTCGGTTGCTTTTTTTATACGGCTGCCAGATAAGGCTTGCAGCATCTGGGCGGCTACCGCTATGATCGGGGCGTTCCTGCAATTCTTAGTGCGAGTATCTGAAAGGGGATACGCGTGGACAGCACGGTCCCTCCGTTTAAGTCTTCTTTCACGTGGC | |
| SCO6666_LA_F | ATATTCTAGATGATGAAGTAGCCGTGGTTG | |
| SCO6666_LA_R | TCTGGTGGCGACGACACGTTCACGGGCTGAACTC | |
| SCO6666_RA_F | TCAGCCCGTGAACGTGTCGTCGCCACCAGAGTC | |
| SCO6666_RA_R | ATATTCTAGACCTCACCAACACGGTCAAG | |
| SCO6666_CRISPR_check_F | AACTCGACGCCCTTGTACTG | |
| SCO6666_CRISPR_check_R | GCATTCTCGGGGTACTGAAG | |
| Oligo used in SCO6666 overexpression | | |
| Oligo | Sequence | |
| SCO6666_XbaI_F | ATATTCTAGAAGCAACGGAGGTACGGACGTGCTGTCCACACTCGCCCG | |
| SCO6666_EcoRI_R | ATATGAATTCTGAAGCCGAGAAAGTCCTTG | |
| SCO1797_Promoter_SacI_F | ATATGAGCTCGCATGACCGAGTACCTGGAC | |
| SCO1797_Promoter_XbaI_R | ATATTCTAGAGCTGTCATGGTAGGTCCGT | |
| SCO1855_Promoter_SacI_F | ATATGAGCTCCGTACAGGCTGGTCAGTGTG | |
| SCO1855_Promoter_XbaI_R | ATATTCTAGAGTGATCGGATACCAAGGGCC | |
| Oligo used in SCO6666 qPCR | | |
| Oligo | Sequence | |
| SCO5820_qPCR_F | CGAGTCCGTCTCTGTCATGG | |
| SCO5820_qPCR_R | ACTGAGTGGCCGGAATCTG | |
| SCO6666_qPCR_F | ACTACGCCCTGCTGATGC | |
| SCO6666_qPCR_R | TCAGTCCGGAGAACAGCAC | |

**References**

1. Chin CS, Alexander DH, Marks P, Klammer AA, Drake J, Heiner C, et al. Nonhybrid, finished microbial genome assemblies from long-read SMRT sequencing data. Nat Methods. 2013;10:563-9.

2. Bonfield JK, Whitwham A. Gap5--editing the billion fragment sequence assembly. Bioinformatics. 2010;26:1699-703.

3. Riemer J, Hoepken HH, Czerwinska H, Robinson SR, Dringen R. Colorimetric ferrozine-based assay for the quantitation of iron in cultured cells. Anal Biochem. 2004;331:370-5.

4. Cobb RE, Wang Y, Zhao H. High-efficiency multiplex genome editing of *Streptomyces* species using an engineered CRISPR/Cas system. ACS Synth Biol. 2015;4:723-8.

5. MacNeil DJ, Gewain KM, Ruby CL, Dezeny G, Gibbons PH, MacNeil T. Analysis of *Streptomyces avermitilis* genes required for avermectin biosynthesis utilizing a novel integration vector. Gene. 1992;111:61-8.

6. Kieser T, Bibb M, Buttner M, Chater K, Hopwood D. Practical Streptomyces Genetics. John Innes Foundation: Norwich, UK, 2000.

7. Anders S, Huber W. Differential expression analysis for sequence count data. Genome biology. 2010;11:R106.

8. Taboada B, Estrada K, Ciria R, Merino E. Operon-mapper: a web server for precise operon identification in bacterial and archaeal genomes. Bioinformatics. 2018;34:4118-20.

9. Arias P, Fernandez-Moreno MA, Malpartida F. Characterization of the pathway-specific positive transcriptional regulator for actinorhodin biosynthesis in *Streptomyces coelicolor* A3(2) as a DNA-binding protein. J Bacteriol. 1999;181:6958-68.

10. Bindea G, Mlecnik B, Hackl H, Charoentong P, Tosolini M, Kirilovsky A, et al. ClueGO: a Cytoscape plug-in to decipher functionally grouped gene ontology and pathway annotation networks. Bioinformatics. 2009;25:1091-3.
